# Lectins as potential tools for cancer biomarker discovery from extracellular vesicles

**DOI:** 10.1186/s40364-023-00520-6

**Published:** 2023-09-29

**Authors:** Md. Khirul Islam, Misba Khan, Kamlesh Gidwani, Kenneth W. Witwer, Urpo Lamminmäki, Janne Leivo

**Affiliations:** 1 Department of Life Technologies, Division of Biotechnology, University of Turku, Kiinamyllynkatu 10, 20014 Turku, Finland; 2 InFLAMES Research Flagship Center, University of Turku, Turku, Finland; 3 Department of Molecular and Comparative Pathobiology, Johns Hopkins University School of Medicine, Baltimore, MD 21205 USA; 4 Department of Neurology, Johns Hopkins University School of Medicine, Baltimore, MD 21205 USA

**Keywords:** Extracellular vesicles, Glycosylation, Lectin, Glycan, Lectin assay, Cancer biomarker

## Abstract

Extracellular vesicles (EVs) have considerable potential as diagnostic, prognostic, and therapeutic agents, in large part because molecular patterns on the EV surface betray the cell of origin and may also be used to “target” EVs to specific cells. Cancer is associated with alterations to cellular and EV glycosylation patterns, and the surface of EVs is enriched with glycan moieties. Glycoconjugates of EVs play versatile roles in cancer including modulating immune response, affecting tumor cell behavior and site of metastasis and as such, paving the way for the development of innovative diagnostic tools and novel therapies. Entities that recognize specific glycans, such as lectins, may thus be powerful tools to discover and detect novel cancer biomarkers. Indeed, the past decade has seen a constant increase in the number of published articles on lectin-based strategies for the detection of EV glycans. This review explores the roles of EV glycosylation in cancer and cancer-related applications. Furthermore, this review summarizes the potential of lectins and lectin-based methods for screening, targeting, separation, and possible identification of improved biomarkers from the surface of EVs.

**Supplementary Information:**

The online version contains supplementary material available at 10.1186/s40364-023-00520-6.

## Extracellular vesicles and glycosylation

Extracellular vesicles (EVs) are mostly sub-micron, lipid bilayer vesicles released by all cell types. EVs allow cells to dispose of unwanted materials and are thought to serve in intracellular communication by facilitating the exchange of genetic materials, proteins, and lipids [1–3]. EVs are greatly heterogeneous, overlapping in physiochemical properties including size. Most commonly, EVs are classified based on biogenesis, with “exosomes” derived from the endosomal system and “ectosomes” (or “microvesicles”) from the plasma membrane. However, these definitions may be of limited value since subtypes of EVs are difficult to identify or separate after release from the cell. Perhaps more importantly, EVs display surface molecules and macromolecules that can potentially be used to identify their cellular source and influence interactions with recipient cells, and this is key to their theragnostic potential.

**Fig. 1:**
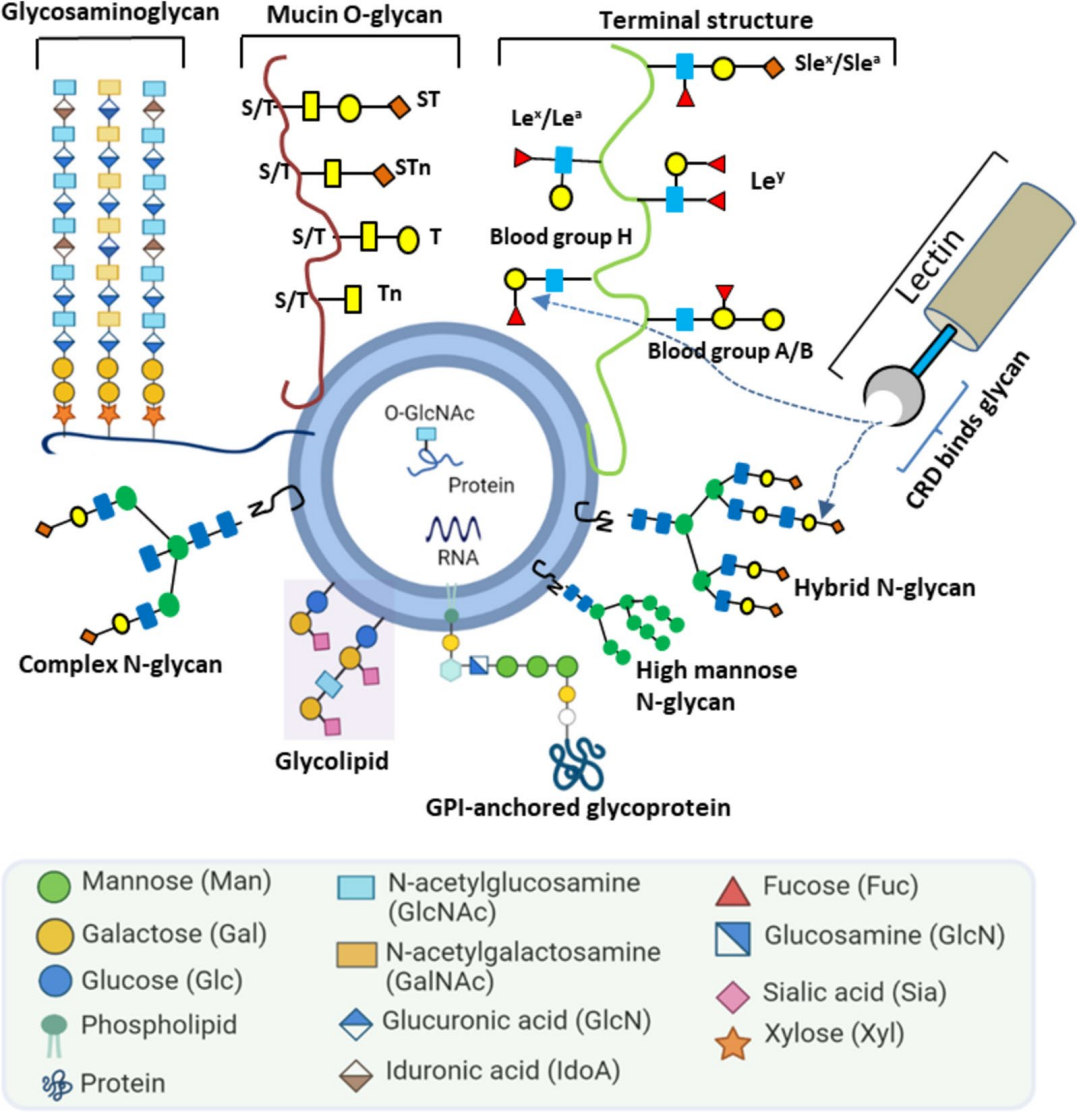
Illustration of glycan structures on the surface of EVs. EVs display various glycoconjugates and alterations of glycan structures are commonly seen in cancers. The CRD (carbohydrate recognition domain) of lectin binds to specific glycans (Herein, GPI = glycosylphosphatidylinositol, sT = sialyl T, sTn = sialyl Tn, sLe^x^ = sialyl-Lewis x, and sLe^a^ = sialyl-Lewis a)

The surface of EVs is highly enriched with glycosylations [4–6]: the covalent attachment of one or more sugar residues to proteins or lipids (Fig. 1). Protein N-and O-glycosylation are the most abundant post-translational modifications in the extracellular milieu [7, 8].

In N-linked glycosylation, a glycan, built on a common core pentasaccharide, is attached to an asparagine residue´s nitrogen atom [9]. Based on the additional sugar moieties attached to the core, the N-glycans can be broadly classified as: a) a high-mannose oligosaccharides that contain mere unsubstituted terminal mannose residues attached to the core; b) hybrid glycans having both mannose and *N*-acetylglucosamine (GlcNAc) residues; c) complex oligosaccharides that lack additional mannoses but contain multiple other sugar types such as fucose, galactose and *N*-acetylneuraminic acid. In contrast, O*-*linked glycosylation involves glycan attachment to side-chain residues of serine, threonine, or hydroxyproline via oxygen. The most abundant proteins with O*-*linked glycosylation are mucins. These large multidomain proteins, produced by epithelial cells, are associated with cancer progression, proliferation, and metastasis [10]. Meanwhile, glycolipids contain different types of glycans compared with glycoproteins. Glycosphingolipid and glycoglycerolipid are two prominent glycolipids, typically conjugated to ceramide and diacylglycerol, respectively. Apart from the glycoproteins and glycolipids mentioned above, other types of glycosylation are also observed in mammalian cells, such as proteoglycans and glycosylphosphatidylinositol (GPI). Proteoglycans are heavily glycosylated and covalently attached to one or more glycosaminoglycan (GAG) chain(s). Each GAG consists of repeated two-sugar units (disaccharides) and has different degrees of sulfation. Based on their core disaccharide units, GAGs are primary classified into four groups including heparin/ heparan sulfate, chondroitin sulfate/ dermatan sulfate, keratan sulfate, and hyaluronic acid [11]. GPIs anchor proteins to the cell membrane. GPIs consist of two fatty acid tails that are inserted into the membrane and linked to a head group, a series of four saccharides, and a phosphoethanolamine residue that links to the carboxyl end of the protein.

Of the different types of EV glycosylation, glycoproteins are the most heavily studied. EV-glycoproteins include integrins (ITGs), mucins (MUC-1, -4, -16), epithelium cell adhesion molecules (EpCAM), carcinoembryonic antigen (CEA), carbohydrate antigen (CA 19–9), cell adhesion molecules such as CD24, and more [12–18]. In the current literature on EV glycosylation, only a limited number of studies have examined glycolipids and proteoglycans. In one EV glycolipid study, Llorente et al. identified several glycolipid candidates on PC3 prostate cancer cells by lipidomic analyses [19]. Proteoglycans are mostly studied in the context of EV biogenesis and cellular attachment [20–22].

Since cellular glycosylation changes can be reflected on released EVs [23, 24], quantitative or qualitative changes to EV glycosylation could form the basis for disease diagnosis [12, 25–28]. Various N-glycosylated EV proteins have been proposed as potential cancer biomarkers [29–31], among them CEA as a marker for colorectal cancer diagnosis and monitoring [32]. ADAM10 and CD109 are also heavily N-glycosylated, with high-mannose glycans that can be used for EV detection [29, 30]. Many other N-linked glycosylation profiling studies have been conducted for the discovery of glyco-specific cancer biomarkers on EVs surface [33, 34]. Similarly, O-linked glycosylations are also common on the EV surface [35, 36]. Glycoprofiling of serum EVs in pancreatic cancer showed a significant elevation of O-glycosylation [35]. Level of O-glycosylated proteins mucin 16 (CA125) was found to be significantly higher in serum EVs of ovarian cancer patients [13]. However, overexpression of mucin O-glycan along with high abundance of sialylated counterparts T, sialyl T, and sialyl Tn antigens is a common trend of glycosylation in cancers [37, 38]. For example, elevated expression of sialyl T, and sialyl Tn antigen could be used as biomarkers for ovarian and gastric cancers, respectively [37, 39]. Similarly, O-GlcNAcylation, *i.e.,* attachment of a single *N*-acetylglucosamine moiety, was found to be elevated on EVs associated with colorectal [36] and breast [40] cancers.

The methods highlighted for analyzing and characterizing EVs glycosylation are mass spectrometry (MS), liquid chromatography (LC), and lectin-based affinity approaches [30, 41–43]. The MS-based methods include matrix-assisted laser desorption-mass spectrometry (DALDI-MS), electrospray ionization-mass spectrometry (EIS-MS), and tandem- mass spectrometry (MS/MS) to detect the structure of a certain glycan. Similarly, chromatographic methods include high-performance liquid chromatography (HPLC) and gas chromatography (GC) for glycan analysis. By getting the advantages of LC–MS/MS and MALDI-TOF–MS techniques, several studies have revealed the detailed structure of glycoconjugates on EVs-derived from cell lines and human biofluids [44–47]. By using these MS and chromatographic methods researchers can gain valuable insights into the composition and structure of EV-glycans (reviewed in [6, 48, 49].

In several studies, after investigation of EV-glycan structures by LC–MS/MS and MALDI-TOF–MS and further validation was conducted by lectin-based techniques [37, 42, 43, 50]. In contrast to MS and LC, lectins can bind and recognize specific glycan motifs without the need for glycan release from samples, liberation or labeling of glycans [51]. Most of the published EV glycan studies have used lectins to detect glycans on the surface of EVs originating from either cell lines or biological fluids [18, 52]. Lectins are carbohydrate binding proteins that recognize specific glycans through their major carbohydrate-recognition domains (CRDs) (Fig. 1). As examples of studies that used lectins, Freitas et al. compared four separation techniques, reporting a diverse set of glycoconjugates on the resulting EVs [37]. They found that lectins E-PHA and L-PHA bind to cancer-related N-glycans, while lectin AAL binds to fucose glycans. In another comparative study, lectin/immune-transmission microscopy (TEM) and ion-exchange chromatography (IEC) were applied to detect differential surface display of sialylated and mannosylated glycan moieties on seminal prostasome EVs of normozoopermic versus oligozoospermic men [41]. Similarly, a study by Surman et al*.* showed that a panel of lectins can bind specific glycan epitopes on EVs compared to that of the parental cell membranes [53]. In this review, we describe different lectin families, including their structures and CRDs. We review the different sources of lectins, including plants, human and recombinant lectins (Table 2). Moreover, we delineate potential approaches whereby lectin-glycan interactions can be used for the separation of EVs and detection of EV glycans that may serve as diagnostic and prognostic biomarkers.

## Cancer biomarker discovery from extracellular vesicles

**Fig. 2:**
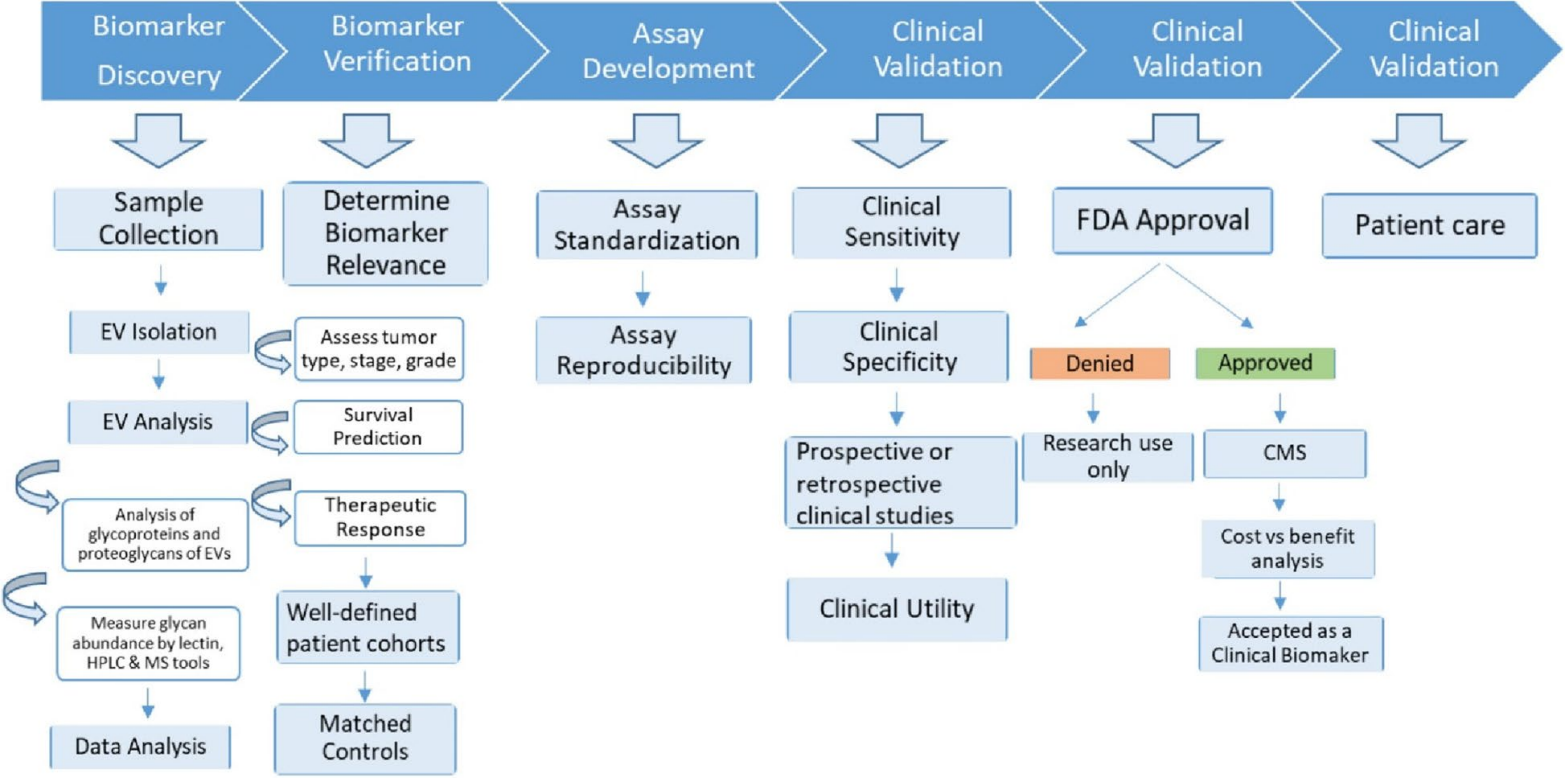
Model flowchart of biomarker discovery using EVs derived from body fluids

Novel cancer biomarkers are urgently needed, not least as integrated components of precision and personalized medicine [54]. Several purposes of biomarkers can be envisioned: 1) diagnosis, or detection of cancers, from early to recurrent; 2) prognosis, to anticipate the likely course of disease; 3) personalization, to assign the right therapies to the individual patient; and 4) monitoring, to assess progression of disease and/or response to therapies. Cancer biomarkers can be protein, DNA, RNA, lipids, carbohydrates, or metabolites which may be changed quantitatively and/or qualitatively during disease. Despite intensive research, only limited numbers of clinically useful biomarkers have been approved by the US Food and Drug Administration (FDA) due to poor sensitivity and specificity. EVs are currently being studied to overcome these limitations, possibly providing novel targets for biomarker discovery (Fig. 2).

EVs are known to occur in different biofluids including urine, blood, saliva, milk, semen, cerebrospinal fluid, and lymph [55]. Secretion of EVs has been found to be higher in cancer patients compared with normal conditions [56, 57]. Similarly, glycosylation patterns of EVs can be different in cancer and non-cancer sources [48]. Some examples of the association between EV glycans or glycoproteins moieties and cancers include: glypican-1(a proteoglycan) [58] and CD133 (prominin-1) [18] for pancreatic; a wide range of N-glycans (bisected, complex, and branched) for prostate, melanoma, and pancreatic [34, 53, 59, 60]; leucine-rich α-2, and α-2-HS-glycoprotein as well as MUC1 for non-small cell lung cancer [61–63]; and LGALS3BP, CD24 and EpCAM for ovarian cancer [15–17].

Starting several decades ago, FDA-approved cancer biomarkers, albeit small in number, have been successfully used in clinics for monitoring, diagnosis, and prognosis of different cancers. Most of these cancer protein markers are either N-or O-glycosylated proteins. Interestingly, the majority of these FDA-approved markers are also found on the surface of EVs derived from different cancers (Table 1).

**Table 1:** EV-associated FDA-approved glycoprotein biomarkers for cancer

FDA approved cancer biomarker used for clinical application						Same biomarker found on EVs			
Biomarker	**Cancer type**	**Sample**	**Glycosylation type**	**Clinical use**	**Year of FDA approval**	**Cancer type**	**EV sample type**	**Publication year**	**Reference**
Alpha-feto protein (AFP)	Testicular	Serum, plasma, amniotic fluid	N-glycosylated	Management of cancer	1992/2008	Melanoma, prostate	Cell lines	2015	[64–66]
CA 15–3 (MUC1)	Breast	Serum, plasma	N- and O-glycosylated	Monitor disease response to therapy	1997	Breast, prostate, bladder	Cell lines, milk, saliva, urine	2004/2007/2009	[67–69]
CA 19–9	Pancreatic	Serum, plasma	Glycan sialyl-Lewis^a^	Monitoring disease status	2002	Pancreatic	Plasma, serum	2017/2020	[70–72]
CA-125 (MUC16)	Ovarian	Serum, plasma	N- and O-glycosylated	Monitoring disease progression, response to therapy	1997/2011	Bladder, breast, colorectal, kidney, melanoma, ovarian	Cell lines, breast milk, serum, ascites, urothelial cells	2009/2011/ 2012/2013/ 2014/ 2016/2017	[73–75]
Carcinoembryonic antigen (CEA)	Colorectal and others	Serum, plasma	N-glycosylated	Aid in management and prognosis	1985	Colorectal, lung	Cell lines, ascites, plasma, urine	2002/2005/ 2009/2011/ 2012/2013/ 2016/2017	[76–78]
Circulating Tumor Cells (EpCAM, CD45, cytokeratins 8, 18 + , 19 +)	Breast	Whole blood	N-glycosylated	Prediction of cancer progression and survival	2005	Colorectal, ovarian, prostate, kidney, lung	Serum, ascites, urine, thymus, B & T cells, breast milk,	2001/2002/ 2005/2007/ 2009/2011/ 2013/2015/ 2017/2018	[79–82]
Estrogen receptor (ER)	Breast	FFPE Tissue	O-GlcNAc	Prognosis, response to therapy	1999	Breast	Urine	2013	[80]
Human epididymis protein 4 (HE4)	Ovarian	Serum	N-glycosylated	Monitoring recurrence or progression	2008	Colorectal, ovarian	Cell lines, seminal plasma, urine	2009/2013/ 2014/2015	[83–85]
HER2/NEU	Breast	FFPE Tissue	N-glycosylated	Assessment for therapy	1998	Breast, colorectal, ovarian, lung, prostate	Cell lines, ascites, breast milk, serum, urine	2002/2005/ 2009/2010/ 2012/2013/ 2016/2018	[75, 77, 86]
Prostate-specific antigen (PSA)	Prostate	Serum	N-glycosylated	Screening, diagnosis and monitoring	1986/1994/2012	Prostate, bladder	Seminal fluid, urine, colorectal cancer cells	2003/2008/ 2009/2012/ 2015/2018	[83, 87, 88]
Progesterone receptor (PR)	Breast	FFPE Tissue	No glycosylation	Prognosis, response to therapy	1999	Breast cancer	Mesenchymal stem cells	2009	[89]
Nuclear mitotic apparatus protein (NuMA, NMP22)	Bladder	Urine	O-linked glycan	Diagnosis and monitoring disease	1996	Bladder, colorectal, lung, ovarian, prostate	Thymus, urine, urothelial and mesenchymal stem cells	2008/2009/ 2011/2012/ 2014/2015/ 2017/2018	[74, 82, 90]
Thyroglobulin (Tg)	Thyroid	Serum, plasma	N-glycosylated	Monitoring disease	1997	Ovarian	T cells, cerebrospinal fluid, ovarian cancer cells	2013/2014/2018	[84, 91, 92]

## Lectins and their potential role in EV research

### Lectins: discovery and basic properties

Lectins are a diverse group of non-antibody glycan-binding proteins (GBPs) that are found abundantly in nature. (The other group of carbohydrate binding proteins, GAG-binding proteins [93], is not addressed here). Lectins were first discovered in plants by Peter Stillmark, who described them in his 1888 doctoral thesis at the University of Tartu, Estonia (reviewed in [94]. While working with extracts from castor bean seeds, he obtained a protein preparation that was capable of agglutinating animal red blood cells. Over time, seeds of many plants were found to contain such proteins, first dubbed “agglutinin”, and later renamed as “lectin”. The terminology ‘lectin’ came from the Latin term ‘legere,’ meaning to select or to choose [95]. Initially, it was thought that lectins were limited to plant agglutinins [96]. However, discoveries of lectins from animals and microorganisms including bacteria, algae, and fungi [97–99] have led to the understanding that lectins are present in all glycan-synthesizing organisms. All lectins are agreed to have exceptional sugar-binding abilities [100] and to play important roles in biological recognition phenomena at the cellular and molecular levels [101, 102]. Today, lectins are defined in that they “should have a carbohydrate recognition domain and should not modify the binding carbohydrates” [103, 104]. Lectins are used as reagents for the detection, isolation, and structural studies of glycoproteins [94]. So far, more than 100 plant and mammalian lectins have been used for the detection, enrichment, or characterization of EVs (Supplementary Table 1).

### Classification of lectins

**Fig. 3:**
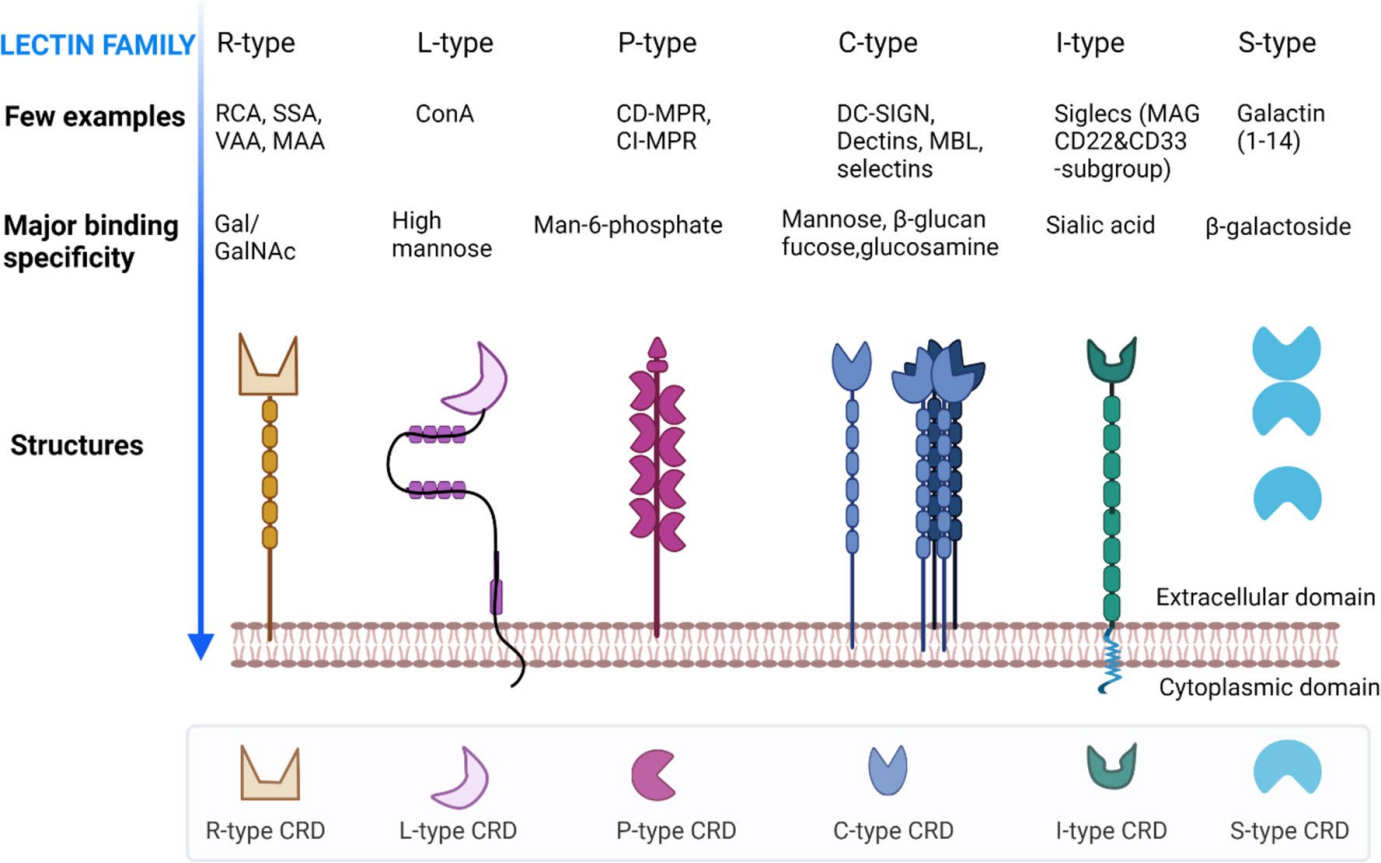
Six lectin families with representative examples and corresponding glycan-binding sites, CRDs, and structures. Different color CRDs represent variation among six lectin families. The number of domains attached with CRD varies among the lectin members. Figure created using BioRender.com

Lectins are divided into several families based primarily on binding specificity for specific glycans [104–106], such as but not limited to GalNAc/*N*-acetylgalactosamine, GlcNAc, mannose, sialic acid, fucose, galactose, T antigen, and novel sialyl lewis antigen (Table 2). Specificity is in turn conferred by carbohydrate recognition domains (CRDs) [107], which can consist of either a single or multiple protein subunits. The number of domains attached to CRDs varies among the lectin families: R-, L-, P-, C-, I-, and S-types (Fig. 3).

**Table 2:**
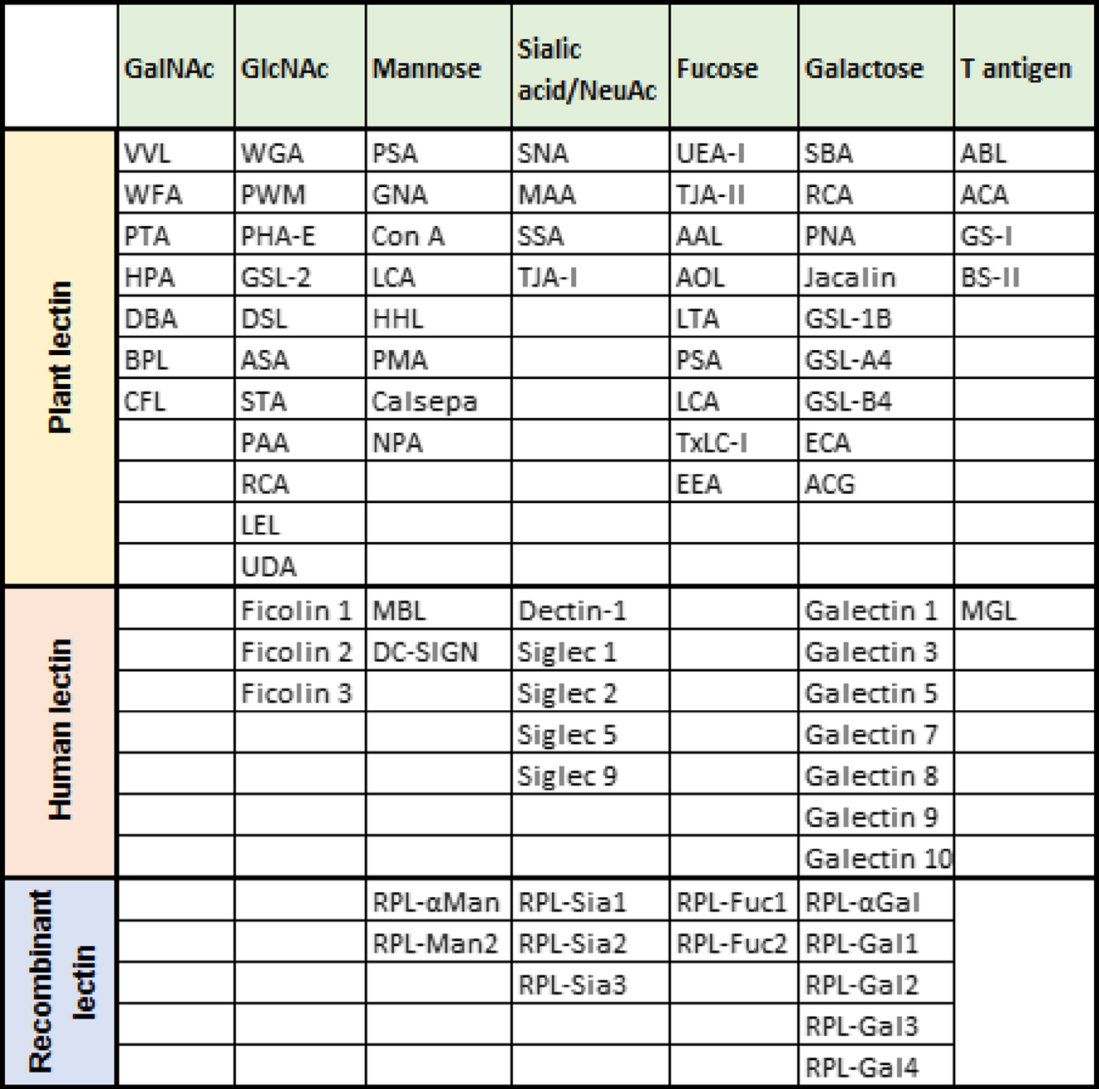
Classification of lectins based on glycan epitope

Several lectins have already been identified on EVs according to the databases Vesiclepedia [108] and ExoCarta [109]. Endogenous tumor lectins are considered as a new class of tumor markers, as they are differentially expressed in tumor vs normal tissues [110]. Moreover, EV-associated lectins play important roles in identifying glycans during tumorigenesis and pathophysiological conditions [111, 112]. However, a wide range of lectins of these families has been used in EV glycosylation detection and profiling (Table 3). Therefore, we now focus on the formation, binding specificity, and biochemical properties of these lectin families below.

**Table 3:** Lectin-based studies of EVs

Lectin used to detect glycan	Glycan on EV	EV source	Separation method	Glycan profiles/other readout	Profiling method	Ref
PNA, MPA, EEA, MAL-I, MAL-II, AIA, STA	Tn antigen and α2,3-linked sialic acids	Healthy donor urine (uEVs)	IA and UC	Lectin- and antibody-captured uEVs show variations in size and surface glycans	Lectin microarray	[113]
WGA, ECL, AAL, PHA-E, WFA, PNA, ConA, SNA, MAL	T-antigen, N-glycan with bisecting GlcNAc and LacdiNAc structure, and α2,3-linked sialic acids	OVMz ovarian cancer cells	UC	Identified specific glycosignature and blocking of those glycosignature has impact on EV composition	Lectin blotting	[39]
ConA, SNA, MAL	LGALS3BP, complex N-glycans of the di-, tri-, and tetraantennary type with fucose, mannose, and bisecting GlcNAc	SKOV3 ovarian cancer cells	UC	EV internalization by recipient cells	Lectin analysis/blotting	[114]
				Sialoglycoproteins & N-glycans found on the EV surface	Lectin blotting	[15]
PHA-M, ConA	Mannose, GlcNAc, GalNAc, and galactose	PCa cells and urine samples (uEVs)	Lectin agglutination	Lectin-induced agglutination of uEVs shows upregulation of PCa-associated mi-RNA	Lectin-immunoaffinity	[115]
WGA, LEL, STL, RCA, BPL, DSL, CAL, DBL, WFL	GlcNAc, and LacNAc oligomers	Healthy donor urine (uEVs)	IA and UC	lectin-based separation of uEVs shows a simple way of biomarker discovery	Lectin microarray	[51]
A panel of 47 lectins	N-glycans, and terminal sialic acids	Murine hepatic cell lines AML12 and MLP29	UC	Role of surface glycans in EV uptake	Lectin microarray	[116]
AIA, PNA, MPA, ABL, RCA-1	Core fucose and α2,3-linked sialic acids	Polycystic kidney disease (PKD) and healthy donor urine (uEVs)	UC and CF	uEVs glycoprofiling shows distinct patterns in PKD vs healthy individual	Lectin microarray	[117]
AAL, WFA, MAA, SNA	α2-3-linked sialic acids, and core fucosylated N- glycan	Cell lines of human HEK293 and glioma H4, and mouse glioma Tu-2449	UC and CF	structure elucidation and validation of N-glycan of EVs	Lectin blotting	[50]
Gal-1, DSL, ConA, AIA, GNA, NPA, PSA, UDA, HHL, CVN, SVN, GRFT, WGA	High-mannose, LacNAc, complex N-glycans, and fucose	H9, SupT1, Jurkat-Tat-CCR5 cells	UC	Glycomic profiling of HIV, cell membrane, and EVs derived from T-cells	Lectin microarray	[118]
A panel of 74 lectins	High mannose and complex N-linked glycans, polylactosamine, and α-2,6-sialic acids	SkMel-5, HT-29, HCT-15, H9, SupT1, Jurkat-Tat-CCR5, and breast milk	UC	Analyzing conserved glycan patterns of EVs	Lectin blotting and lectin microarray	[34]
PHA-M, ConA	Mannose, GlcNAc, and GalNAc	Blood, urine, ascites, and pleural liquids	UC and lectin agglutination	Lectin-aggregated EVs separation approach and characterized those EVs through proteomic studies	Lectin-induced agglutination, lectin blotting	[119]
GNA, DSA, PNA, SNA, MAA	Oligomannose	Astrocyte-enriched primary cultures (mouse)	UC	Synapsin is an oligomannose-binding lectin and releases from glial-derived EVs	ELISA, lectin staining	[120]
A panel of 45 lectins	α2-3 and α-2,6-linked sialic acids, fucose, and mannose	ADSC cells	UC	Glycan profiling of EVs and analysis of their uptake in vivo	Lectin microarray	[121, 122]
				Specific glycan biomarker discovery for osteogenic differentiation		
SSA	α-2,6-linked sialic acid	Melanoma cells	UC	Studied EV biogenesis and also identified membrane proteins on EVs as a membrane marker using a lectin	Lectin blotting	[123]
OAA	High mannose	Human glioblastoma, melanoma, colon and lung cancers	UC and lectin agglutination	A mannose binding lectin is used for affinity isolation of tumor derived EVs	Lectin-affinity approach	[30]
MAL-II, SNA, Jacalin, PNA, RCA120, GSA I-B4, DBA, SBA, Con A, sWGA, GSA-II, PSA, UEA-I, LTA	Galβ1, 3GalNAc, high mannose, and α-2,6-linked sialic acid	eAMCs	UC	Comparison of glycan composition on eAMCs and eAMCs-EVs	Lectin array	[124]
PHA-E, PHA-L, AAL	complex N-glycans with β1,6-branched tetraantennary and bisecting GlcNAc, and fucosylation linked (α1-3) or (α1-6)	MKN45 and glycoengineered MKN45 gastric cancer cell lines	UC, TEI, ODG, SEC	Different isolation approach yields distinct glycan associated EVs populations	Lectin blotting	[37]
SNA, ConA, Gal-3	Sialylated and mannosylated glycan structure	Seminal plasma from normozoopermic and oligozoospermic men	UC and gel filtration	Comparative analysis of EVs focusing their glycosylation	Lectin-TEM, and ion-exchange chromatography (IEC)	[41]
A panel of 45 lectins	Glycosylation of CD133 by sialic acids	Ascites samples and cell lines	TEI	Heavily glycosylated CD133 on EVs could be used as a potential biomarker for advanced pancreatic cancer	Lectin microarray	[18]
MAA, SNA, GNA, AAA, PHA-E and PHA-L	α-2,3 and α-2,6-sialic acids, fucoses, complex N-glycans with β1,6-branched tri/tetraantennary and bisecting GlcNAc	Primary WM115, WM793 and metastatic WM266-4, WM1205Lu melanoma cells	UC	EVs have specific glycan composition compared with their parental cell membrane fraction	Lectin-blotting	[53]
A panel of 48 lectins	High mannose, Galβ (1 → 3)-GalNAc, LacNAc, and GlcNAc	Adult helminth pathogen* F. hepatica*	UC	EVs released by parasite can interact with recipient host cells	Lectin microarray	[125]
SNA, Jacalin, PNA, HPA, UEA-I	Siaα-2,6Gal/GalNAc, fucose, T and Tn antigens	PANC-1 and HeLa cells	UC	Multiplexed detection of EV glycan pattern	Lectin array	[38]
A panel of 96 lectins, lectin rBC2LCN assay specific to EVs	Fucα1-2 Galβ1-3 GalNAc, Siaα-2,6 and LacNAc	hiPSCs and non-hiPSCs	Magnetic bead-based IA approach	Glycan profiles of EVs derived from stem cells	Lectin microarray and sandwich assay	[126]
A panel of 45 lectins	Galα1-3 GalNAc (T-antigen), and Siaα-2,3 Galα1-3 GalNAc (sialyl T-antigen)	Pancreatic cancer patient serum	Magnetic bead-based IA approach	Differential glycomic profiles of EVs and recognition of O-glycosylated EVs for pancreatic cancer detection	Lectin microarray	[35]
DBA, ECA, PHA-E4, MAM, WGA, ConA	GalNAc, GlcNAc, high-mannose, and Siaα2-3Gal	MDAMB231luc—D3H1 &-D3H2LN, and BMD2a breast cancer cells	UC	Identified altered glycosylation patterns on EVs which has inhibitory effect on EVs uptake	Lectin blotting	[127]
PHA-E	N-glycans with bisecting GlcNAc	MCF10A, MCF7, SKBR3, and MDAMB23 cell lines	UC	Modification of bisecting GlcNAc level on EVs has significant effect on metastasis of recipient cells	Lectin histochemistry	[128]
ConA, WGA, RCA, SNA, MAA, PHA-E, PHA-L, LCA, PNA, VVL, DBL	High-mannose, Siaα2-3Gal, and GlcNAcβ1.4	Human urine (uEVs)	UC	uEVs isolation from THP-depleted urine and use those uEVs for lectin-EV assay	Lectin-EV binding assay	[129]
AAL, SNA, PHA-E, PHA-L	Bisected and branched N-glycans, Fucα1-6GlcNAc, and α2,6-linked sialic acid	B16F10, Pan02, 4T1, AsPC1, MADAMB4175 cell lines	Asymmetric flow field-flow fraction (AF4)	Identification of EVs and their subpopulation	Lectin blotting	[43]
A panel of 34 lectins	Fucα1-2Gal, GalNAc, GlcNAc, mannose, and sialic acid	PCa cell line and bladder cancer patient urine (uEVs)	UC	Integrin-lectin assay significantly discriminate bladder cancer patients compared to benign control	Lectin-nanoparticle assay	[12]
SNA, ConA, AAL	α2,6-linked sialic acid, Fucα1-6GlcNAc, and mannose	Pancreatic cell line and patient serum	UC	Lectin conjugated janus nanoparticles (JNPs) specifically binds to glycans on pancreatic cell derived EVs and shows diagnostic potential	JNPs assisted- lectin-EV assay	[130]
A panel of 50 lectins	High-mannose, GlcNAc, GalNAc, and sialic acid	Helminth parasite *F. hepatica*	UC	Parasite EVs has influence on host-immune response during infection and glycosylated proteins of these EVs play role in EVs internalization	Lectin microarray	[42]

*UC* ultracentrifugation, *IA* immunoaffinity, *CF* centrifugal filtration, *SEC* size exclusion chromatography, *TEI* total exosome isolation kit, *ODG* optiPrepTM density gradient, *ADSC* adipose derived mesenchymal stem cells, *eAMCs* Equine amniotic mesenchymal cells

GlcNAc = *N*-acetylglucosamine, GalNAc = *N*-acetylgalactosamine, Gal = galactose, Tn = T antigen, sTn = sialyl-Thomsen-nouveau antigen, LacNAc = *N*-acetyllactosamine

#### R-type lectins

R-type lectins contain an R-type carbohydrate recognition domain (CRD) that is structurally similar to the CRD in ricin. Ricin is known as the first lectin, discovered in *Ricinus communis* (castor bean) [94]. The R-type lectins usually recognize Gal/GalNAc moieties and occur in plants, bacteria, and animals [131]. Examples include *Ricinus communis* agglutinin (RCA-I & II), *Viscum album* agglutinin (VAA), *Sambucus sieboldiana* agglutinin (SSA), *Sambucus nigra* agglutinin (SNA), and *Maackia amurensis* agglutinin (MAA-I&II), which have been used in EV glycome studies [114, 124].

#### L-type lectins

The L-type lectins are typically found in the seeds of leguminous plants and require Ca^2+^ ions for ligand binding. This group of lectins is distinguished from other types by tertiary structure, consisting of antiparallel β sheets joined by short loops and β-bends and devoid of α -helices [132]. Examples of L-type lectins include concanavalin A (ConA) and phytohemagglutinin E (PHA-E), which are also potential tools for studying EV glycosylation [115, 128].

#### P-type lectins

There are two members of the P-type family: cation-dependent mannose 6-phosphate receptor (CD-MRP) and cation-independent mannose 6-phosphate receptor (CI-MRP), which can specifically recognize phosphorylated mannose residues [133]. Members of this group typically bind to mannose 6-phosphate (M6P), with the phosphate group being key to high carbohydrate binding affinity. The P-type lectins bring the M6P signal to lysosomes within the cells, resulting in the generation of functional lysosomes [134].

#### C-type lectins

C-type lectins are usually found on animal cells, including immune cells such as macrophages and dendritic cells. C-type lectins require Ca^2+^ ions for ligand binding and share primary and secondary structural homology in their CRDs. The CRD of C-type lectins is composed of a compact region of 110–130 amino acid residues with a double loop and two antiparallel β-sheets. Members have one or more characteristic C-type lectin-like domains (CTLDs) and have been subdivided into 17 subfamilies based on domain orientation and phylogeny [135]. Several subfamilies are again sub-classified based on common carbohydrate motifs. C-type lectins have a diverse range of functions, including cell-to-cell interaction, immune response to pathogens, and apoptosis [135]. Examples of these lectins include DC-SIGN, MGL, MMR, MBL, and selectins, which have been reported for the characterization of EVs in several studies [136–138]. Furthermore, some C-type lectins have been discovered in EVs [55], such as selectins (subtypes P, E, and L) and collectin (collectin-12) [55, 139–141].

#### I-type lectins

I-type lectins are members of the immunoglobulin superfamily (IgSF) that typically recognize sialic acids and other carbohydrate ligands. Most members are siglecs, which are type I transmembrane proteins. Siglecs are divided into two groups: one group includes siglec1 (CD22 molecule), siglec4 (MAG), and siglec15, and the other includes siglec3 (CD33 molecule), siglec5-11, and siglec14 [142, 143]. Siglecs are well known for their functional roles in cell–cell adhesion and cell signaling [144]. Because of their specificity for sialic acid, which is highly expressed on certain cancer EVs [15, 34], siglec lectins might be used to detect cancer glycosylations [145] and EV biodistribution [146–148].

#### S-type lectins (galectins)

S-type lectins specifically recognize β-galactosides with their CRDs and are thus also termed galectins. Galectins are anchored on cells through the interaction of CRDs and cell-glycoconjugates. Galectins are found not only in mammals but also in birds, fishes, nematodes, sponges, and fungi. Galectins play important roles in immune response, inflammation, and tumor progression and metastasis [149–151]. Several galectin candidates have been identified in EVs derived from cancer cells and biological samples and have been extensively studied in EV research (Table 4). Galectin-1 and -3 have been found in EVs derived from bladder, ovarian, and colon cancer cells [67, 152–154]. Galectin-3 is also found in EVs of human semen [155] and has elsewhere been reported to be involved in polarized EV release [156]. Interestingly, galectin-3 is related to antimicrobial activity against bacteria and fungi [157–159]. Barres el al. reported that galectin-5 on rat reticulocyte EVs was essential for EV uptake by macrophages [112]. Galectin-9 was found on EVs from Epstein-Barr virus-infected nasopharyngeal carcinoma cells [160].

**Table 4:** Galectin-associated EV studies

Lectin	EV source	Separation method	Readout	Method	Ref
Galectin-3	Melanoma SK-Mel-5 cells	UC	Complex N-glycans mediate protein shorting to EVs	Lectin blotting	[161]
Galectin-5	Rat reticulocytes	UC	Galectin-5 has effect on EVs uptake by macrophages	Galectin blotting	[112]
Galectin-3	MDCK kidney cells	UC	Mechanism of galectin-3 delivery into ILVs for exosomes release	Galectin blotting	[156]
Galectin-3	Mice*, C. neoformans* strain, and patient serum	UC	Gal-3 inhibits fungal infection through direct antifungal effect	ELISA	[158]
Galectin-3	Mice*, P. brasiliensis* strain, and patient serum	UC	Gal-3 inhibits fungal growth through multiple mechanism	Galectin blotting	[159]
Galectin-3	Human semen	UC	Expression of gal-3 on human semen EVs	Galectin blotting	[155]
Galectin-9	Epstein-Barr virus (EBV)-infected NPC cells	UC and magnetic bead-based IA	EVs from EBV-infected tumor cells contain gal-9 protein	Galectin blotting	[160]

### Application of lectins in biomarker discovery

Protein glycosylations can be highly useful in biomarker discovery, including in cancers. Glycosylation patterns may be a reliable signal of cancer [162], and several glycobiomarkers have been discovered that might be clinically useful [163]. Indeed, most cancer markers used in clinics are glycoproteins, and their glycan moieties may be structurally different in cancer [164]. Nevertheless, most FDA-approved cancer biomarkers are monitored at the protein level, i.e., antibodies of immunoassays used for the biomarker detection recognize merely protein-based epitopes. Biomarker detection with protein-specific antibodies can be hampered by moderate specificity and sensitivity, the presence of putative marker proteins in healthy and benign individuals, and even by the possibility that glycoconjugates mask protein epitopes in cancer [165]. As a result, lectins are an attractive option for the identification of altered glycosylation patterns in cancer [166]: they have often high sensitivity and relative specificity and are widely available [105]. Glycans of MUC1 (CA15-3), carbohydrate antigen (CA19-9) and alpha-fetoprotein (AFP) are monitored for management of breast cancer, pancreatic cancer, and hepatocellular carcinoma (HCC), respectively [167]. In HCC, elevated AFP is effectively detected by lectin LCA for early diagnosis [168]. Glycoconjugates on EVs may influence EV release, biodistribution, and uptake, and, as noted previously, can also be used for EV detection and separation with the help of lectins (reviewed in [48, 49]. In sum, lectins are valuable tools for EV biomarker detection and discovery.

### Lectin-based advanced methods for studying EVs

**Fig. 4:**
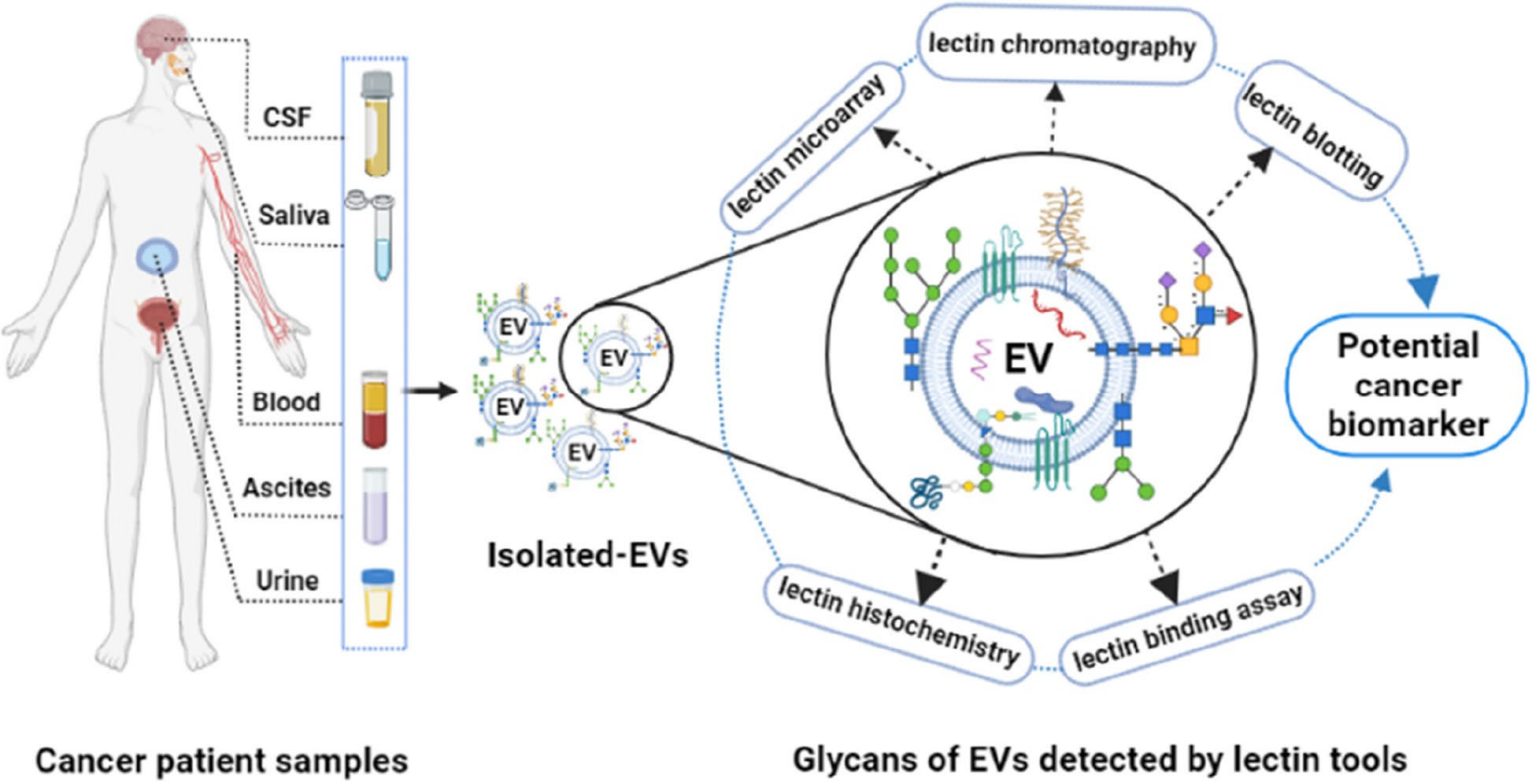
Overview of lectin-based tools for studying surface glycans on various biofluids derived EVs

A wide variety of lectins and lectin-based assays have been used to profile EVs for biomarker discovery (Table 3). In this section, we address several lectin-based approaches, including lectin microarray [34, 117, 118, 169], immobilized lectin affinity chromatography [30, 51, 113, 170], lectin blotting [39, 114], lectin histochemistry [128], lectin-EV binding method [129, 136], and lectin-based immunoassays [12, 136, 171, 172]. In these methods, lectins target specific glycan moieties of EVs, lending insights into altered glycosylation and tumorigenesis and accelerating biomarker discovery (Fig. 4).

#### Lectin microarray for EV glycan profiling

Lectin microarray is a popular technology for medium- to high-throughput glycosylation analysis that was first proposed by Kuno et al. [169] in 2005. Lectin microarrays can be used for rapid and highly sensitive profiling of complex structures in both pure and crude glycoproteins [173], avoiding the liberation of glycans. They may have advantages over conventional methods like liquid chromatography (LC) and mass spectrometry (MS), where long branches and diverse structures of glycans create analytic challenges.

**Fig. 5:**
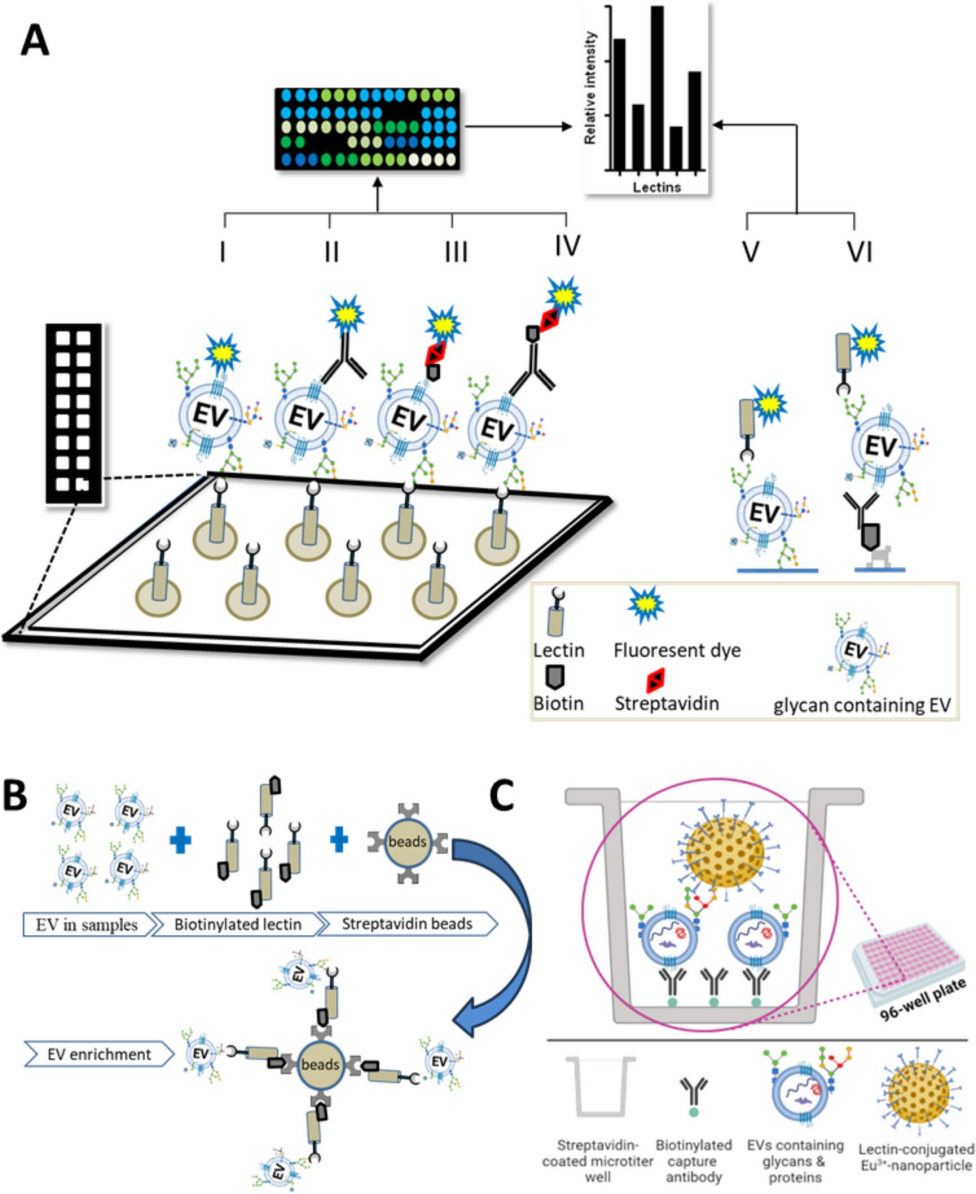
Lectin-assisted approaches for studying EVs. **A** (I-VI) Schematic of lectin microarray approaches. In I-IV, lectins are immobilized onto a surface, e.g., a glass plate. For detection of captured EVs, labels might include I) a fluorescent dye that directly labels the EV, II) a fluorescently labeled antibody to an EV surface antigen, III) fluorescently labeled streptavidin that recognizes biotinylated EVs, and IV) a biotinylated antibody-fluorescent streptavidin system. Systems other than streptavidin–biotin could also be used. In V-VI, EVs are V) directly immobilized or VI) captured by immunoaffinity followed by detection with lectin(s). **B** Graphical representation: separating pure EVs using a lectin-bead technology. **C** Schematic representation of Eu^3+^-nanoparticle based sandwich assay. Biotinylated antibody is immobilized on the surface of streptavidin-coated microtiter 96-well plates for capturing EVs. The captured EVs are detected with lectin-coated fluorescent Eu^3+^-nanoparticle

Several groups have applied lectin microarrays to EV studies, of which several variations are possible (graphically presented in Fig. 5). Basically, surface-immobilized lectins are used either to capture EVs (which are then detected using one or more labeling methods) or to detect specific glycosylations on EVs that have been captured e.g., by EV-surface protein binding antibodies. Recently, Feng et al. described the use of lectin-mediated in situ rolling circle amplification with an EV array for efficient multiplex detection of EV glycan structures [38]. The first report, in 2009 [118], used lectin microarrays to analyze surface glycans of intact HIV-1 virions and EVs from T-cells, finding a common glycome with enrichment and exclusion of specific glycans. A related study from the same laboratory [34] found that EVs have conserved glycan surface signatures, predominantly consisting of high mannose and complex N-linked glycans, polylactosamine, and α-2,6-sialic acids. Several studies have used lectin arrays to profile EVs from complex body fluids. As an example, Gerlach et al. [117] used a 43-lectin microarray to profile isolated urine (uEVs) and a non-vesicular protein fraction containing THP (Tamm-Horsefall protein). Surface glyco-patterns of uEVs were distinct compared with the THP fraction, and binding proficiency of lectins to the THP fraction was limited. Furthermore, uEVs from patients of autosomal dominant polycystic kidney disease (ADPKD) were compared with those of age-matched healthy subjects to seek biomarkers of ADPKD.

##### There are numerous additional examples of lectin microarrays being used for discovery of cell and disease markers

In advanced pancreatic cancer, lectin microarray was used to reveal highly glycosylated CD133 as a prognostic marker on EVs from malignant versus non-malignant ascites [18]. In another study, using serum of pancreatic patients, A 45-lectin microarray identified six lectin candidates, including ABA and ACA, that differentiated O-glycosylated EVs of pancreatic cancer patients from those of the controls [35]. Importantly, ABA- and ACA-positive EVs were detectable in serum even when commercial pancreatic cancer serum marker CA19-9 was negative. Moreover, Bertokova et al. showed the potential of lectin fluorescent microarrays for the analysis of glycans of EVs from prostate cancer cells [174]. Saito et al. found EV glycosylation patterns specific to human induced pluripotent stem cells (hiPSCs) using a panel of 96 lectins [126]. Lectin rBC2LCN bound to hiPSCs-EVs but not to EVs from control cells. Using rBC2LCN in combination with phosphatidylserine receptor Tim4 (rBC2LCN-Tim4), they developed a sandwich assay that was superior to a previous antibody-based assay (Tim4-CD63). Desantis et al. used 14 lectins to characterize amniotic mesenchymal cells and their EVs [124]. To identify markers of therapeutic EVs, Hayashi et al. used a 96-lectin array, reporting that fucose-specific TJA-II distinguished therapeutic MSC-EVs [175].

##### From lectin profiling to function: the case of cellular uptake

Lectin arrays have been used to understand not only EV glycan profiles but also to probe EV biogenesis and uptake. Shimoda et al. isolated EVs from human adipose derived mesenchymal stem cells (ADSC) for glycan profiling using a 45-lectin evanescent-field fluorescent (EFF) array [121]. The greatest signal intensities were associated with polylactosamine-binding lectin (STL, UDA, and LEL), GlcNAc-binding lectin (WGA), and Gal-binding lectin (DSL). Specific siglecs (-1, -2, and -3) preferentially bound to ADSC-EVs (vs corresponding cell membrane) by recognizing sialic acid residues (a2-3, a2-6), suggesting the possibility of sialic acid involvement in biogenesis and cellular uptake. The EFF-lectin array method was also used to compare EVs of osteogenically differentiated and undifferentiated MSCs [122]. Lectins such as BPL, ECA, SBA, and WFA bound more strongly to EVs of differentiated cells, indicating that EV glycans may discriminate cellular differentiation and cancer stages. The same approach was used to establish that EV glycans define EV heterogeneity and influence biodistribution and cellular uptake efficacy [24]. The authors highlighted that glycoengineering of EV could be used to manipulate EV-cell interactions. Williams et al. added support for the role of glycans in EV uptake, comparing EV surface glycans from two murine hepatic cell lines using a 47-lectin microarray [116]. Clos-Sansalvador et al. used a 26-lectin array and PNGase-F treatment to investigate the importance of MSC-EV N-glycans in EV-endothelial cell interactions and EV uptake [176]. Together, these studies emphasize that EV glycans may have functional consequences, opening the door for glycoengineering of EVs and novel therapies.

EVs of non-human organisms have also been studied by lectin microarray. EVs were isolated from the helminth parasite *F. hepatica* [42, 125, 177] and studied with a 50-lectin microarray for the characterization of surface glycan topology. Among the 50 lectins, mannose-binding and complex type N-glycan-binding lectins showed the highest binding intensities. Furthermore, a total of 618 proteins were identified by proteomic analysis, among which 121 and 132 proteins contained putative N-and O-linked glycosylation sites, respectively [42]. These surface glycans thus hold potential for biomarker development in infectious diseases.

#### Lectins for EV separation

EV separation techniques remain a center of attention, with ongoing debate about how these techniques affect our conclusions about EV biogenesis, release, uptake, and roles in disease development. Some traditional techniques for EV separation may be tedious, labor-intensive, and costly, and certain techniques may result in EV aggregation or damage or substantial presence of co-isolates that confound interpretation [178, 179]. For glycosylation studies, legacy separation methods do not yield subpopulations based on glycosylation [37]. To overcome these limitations, lectin-based affinity capture has gained popularity. Echevarria et al. first reported a lectin-based approach in 2014 to separate EVs from urine [51]. Of 62 screened lectins, STL, WGA, and LEL showed significant binding to uEVs, with STL having the highest affinity and also avoiding THP binding. A capture method was then devised, using a biotin-streptavidin and magnetic bead approach (graphically presented in Fig. 5B). A galectin-coupled magnetic bead approach was used to isolate pure EVs from human plasma for head and neck cancer biomarker discovery [180]. Interestingly, Gerlach and colleagues reported that lectin separated EVs had greater purity than EVs prepared by other methods [113]. Another study showed that EVs isolated by lectins (PHA-M and Con-A) from different biological fluids could be used to validate EV compositional studies [119]. Similarly, Ward et al. (mentioned in [113]) proposed uEV isolation by MAL-II, WGA, STA, and LEL lectins, while Samsonov et al. captured EVs with Con-A lectin and performed RNA analysis for prostate cancer diagnosis [115]. Taken together, lectin affinity capture is useful for enriching specific subsets of EVs, including in clinical applications.

Since high mannose-type glycans are highly enriched in tumor-derived EVs [34, 170], the Maruyama group developed a mannose-glycan-based isolation technique for tumor-derived EVs using a high mannose-type glycan-binding OAA lectin [30]. They showed that mannose-binding OAA lectin captures small EVs from different tumor cells, such as glioblastoma, melanoma, and colon and lung cancers. Their findings showed that N-linked glycans allow high-affinity capture of tumor-derived EVs.

Furthermore, Kanao et al. studied EV separation based on their surface glycans and revealed the difference of protein contents in EVs [181]. Interestingly, in their lectin-based EV separation method, apart from using a typical agarose gel, they used a sponge-like monolithic polymer (SPM) which has large flow-through pores that ensure high EV recovery.

#### Lectin blotting

Lectin blotting uses lectins to detect glycosylation on proteins or lipids that have been separated by gel electrophoresis (SDS-PAGE) and transferred to adsorbent membranes. The Costa group used lectin blotting to profile EVs and parent cellular extracts, finding EV enrichment with specific sialic acid and mannose-containing glycoproteins [114]. Sialoglycoproteins that were identified on EVs from ovarian carcinoma cells were subsequently confirmed by lectin blotting [15, 39]. Furthermore, N-glycans from cell lines derived EVs were analyzed by MALDI-TOF mass spectrometry and HPLC and subsequently validated by lectin blotting [50]. Zhang et al. applied asymmetric flow field-flow fraction (AF4) to obtain three size-separated extracellular particle populations including EVs, using lectin blotting to show that these populations displayed distinct N-glycan and sialylation patterns [43]. In another study, Nishida-aoki et al*.* found differential glycosylation patterns on EVs from breast cancer cell lines [127]. Using lectin blotting, they demonstrated that removal of O-and/or N-glycosylation from the surface of EVs has inhibitory effects on EV uptake. Similarly, alteration of complex N-glycans could control the recruitment of specific glycoproteins (e.g.-EWI-2) into EVs [161]. In another study, Tan et al. showed that modification of bisecting GlcNAc can suppress metastasis induced by EVs from breast cancer cells [128]. Using lectin blotting by PHA-E lectin that specifically binds to bisecting GlcNAc, they found that bisecting GlcNAc levels were significantly lower in human breast cancer cells compared to heathy controls [128, 182].

The presence of several cancer-associated glycoprotein biomarkers has been confirmed in various EVs subpopulations [48, 49]. Kondo et al. used lectin blotting by SSA and WGA to identify distinct patterns of N-glycosylation on small EVs of small-cell lung carcinoma (SCLC) and non-small-cell lung carcinoma (NSCLC) cells [183]. They also found a molecular link between lung cancer types and integrin N-glycosylation of small EVs. Recently, blotting with six lectins was done in a study that reported the presence of disease-associated glyco-epitopes in bladder cancer-derived EVs [184]. In another study, after 20-lectin microarray identified altered glycans on EVs from gastrointestinal cancer, findings were validated using lectin blotting [185].

#### Lectin-nanoparticle assays

It is already established that lectin can easily conjugate with nanoparticles, which can be used for targeted detection of biomolecules [186]. Based on this approach, Choi et al. developed nanoparticle assisted microfluidic device that can detect cancer-derived EVs following lectin-glycan interaction, which could discriminate pancreatic cancer EVs from those of the control sources [130]. Particularly, lectins with specific affinity, for sialic acid such as lectin SNA and fucose such as AAL, were attached to bifunctional Janus nanoparticles (JNPs), which facilitated binding to EVs in the microfluidic device. Moreover, lectin-conjugated JNPs successfully captured EVs from pancreatic cells as well as serum samples with high affinities that were comparable to those of anti-CA19-9 antibody. This platform holds the possibility to achieve new biomarker discovery targeting glycan moieties of EVs derived from pancreatic cancer sources.

Our group developed a fluorescent europium nanoparticle (Eu^3+^-NP)-assisted lectin approach for the detection of glycan on EVs. The lectins coated on polystyrene nanoparticles of ca. 100 in diameter and containing ca. 30000 Eu^3+^ ions per particle for time-resolved fluorescence-based detection [136]. Due to the presence of a number of lectins per nanoparticle, NP can give avidity effect in binding which helps to overcome potential issues related to the limited binding affinity of individual lectins [136, 187]. Unlike antigen–antibody binding affinities (dissociation constant, *K*_d_ = 10^–8^-10^–12^ M), lectin-glycan affinities are much lower (*K*_d_ = 10^–4^-10^–7^ M) [188]. Altogether, the NP-aided tool confers a highly sensitive time-resolved fluorescence-based detection in a simple two-step sandwich assay (schematic representation in Fig. 5C), and shows improved performance compared to conventional europium chelate labeled lectin reporters [136]. Furthermore, based on the developed assay platform, we have identified a glycovariant of ITGA3 on urine of bladder cancer (BlCa) patients which could be used for BlCa detection [189]. In a follow-up study, aiming to construct the further improved lectin assay for the specific detection of EVs, we have tested a panel of 34 lectins among which a fucose binding lectin UEA showed strong binding intensities toward EVs. This lectin-nanoparticle assay further was validated with a small cohort of clinical samples from urological malignancies, where we observed that lectin assay could discriminate bladder cancer patients compared to clinically challenging benign prostate hyperplasia and healthy individuals [12]. Following a similar approach, in another study, we tested 27 lectins for the detection of EVs from breast cancer cell culture medium. Among 27 lectins, fucose-binding lectin AAL and UEA showed strong binding to EVs from media of three breast cancer cell lines compared with one control cell culture medium [172].

## Challenges and future directions

The EV field has grown rapidly in the last decade [190], and although significant progress has been made in EV-based cancer biomarker discovery [191, 192], translating these findings into clinical practice (including therapeutics) faces several challenges. These include the technical challenges of EV separation and detection and the need for more insights into molecular mechanisms governing EV release and EV uptake by target cells. In each of these areas, we submit that glycosylation holds a key to progress and that lectin-based approaches, with their specificity and ease of implementation, will unlock doors.

EV surface glycosylation patterns are a veritable hidden treasure for the discovery of disease biomarkers and disease drivers. Progression and development of cancers in particular are associated with aberrant glycosylation, and these altered patterns are also transmitted on cancer derived EVs [48]. In this review, we have examined the utility of lectin–based tools and strategies to identify EV glycovariant markers in cancer and beyond. A good number of innovative lectin-based methods have already been reported for studying glycosignatures of EVs (Table 3). We provide a comparative summary of advantages and disadvantages of lectin-based detection approaches **(**Table 5). Because of the wide variety of lectins that are available, there is also the possibility of using a combination of markers when one does not suffice [193].

**Table 5:** Advantages and disadvantages of lectin-based detection approaches in cancer biomarker discovery

Tools used	Advantages	Disadvantages
Lectin arrays	▪ rapid and highly sensitive ▪ allow high throughput screening ▪ allow multiplexing ▪ do not require sample preparation ▪ use small amount of samples ▪ unbiased binding to glycans	▪ non-specific binding may happen ▪ need extensive optimization
Lectin blotting	▪ easy to use ▪ reproducible ▪ highly sensitive and specific ▪ suitable for complex protein samples ▪ visualize small amounts of antigens	▪ membrane may affect chemical stability of lectins ▪ non-specific binding may happen
Lectin affinity chromatography	▪ produce pure analyte ▪ high affinity ▪ do not require sample preparation ▪ do not need purified glycans	▪ may co-elute other proteins ▪ need large amounts of samples ▪ non-specific binding may happen ▪ time-consuming
Lectin-binding assay	▪ simple and rapid ▪ very cost effective ▪ easy to perform ▪ requires minute amounts of samples ▪ high sensitivity and specificity	▪ purified glycan may require as a standard ▪ non-specific binding may happen

Though lectins effectively recognize and bind the fine glycan structures, they cannot provide sufficient information regarding the glycan components and glycan types such as specific monosaccharides and linkages present in the glycan chain [194]. Some lectins have overlapping binding affinities and specificities towards multiple glycan structures which lead to difficulties in precisely identifying the glycan structures of interest [195, 196]. It is also known that the recognition of lectins to glycan structures depends on the appropriate orientation of glycans. Changes in the glycan conformation may also influence the lectin binding which leads to false-positive or false-negative results. Sometimes, glycan structures can be masked or hidden by other glycans or biomolecules which may prevent lectins from binding to the target epitopes [197]. Additionally, the binding of lectins towards glycans may be influenced by the surrounding microenvironment such as temperature, presence of ions, and pH [198]. This factor may affect the robustness and reproducibility of lectin-based assays. Despite these limitations, lectins and lectin-based methods remain valuable tools in glycan research. To overcome some of these challenges, scientists often use lectin tools in combination with other techniques such as MS and HPLC for a better understanding of detailed glycan structures and their applications. Moreover, advances in synthetic glycobiology and glycan array technology are constantly improving our ability to study glycan interactions and their functions in a systematic way.

To ensure accurate and reliable results from lectins and their carbohydrate interaction, it is essential to verify this interaction through a) competitive inhibition, b) through modification of the carbohydrate structures (i.e., by oxidation or enzymatic means) or c) by comparison with lectins which have been modified to disrupt their carbohydrate-binding domains [34, 117, 199]. This combination approach is crucial when studying lectins and their biological roles, as it helps to avoid false positive or negative results and provides concrete evidence for carbohydrate-mediated binding [199, 200].

Several studies have reported that glycans found on EVs are to some extent different compared to their parental cell membrane-associated glycans [53, 118]. In another study, as expected, glycan profiles of plasma-derived EVs are distinct from donor-matched whole plasma [201]. However, we have found limited studies where comparisons between glycans on EVs vs tissue samples are addressed. In a recent study, glycan-associated proteins such as CD147, BGN, VCAN, and TNC were found to be enriched in tumor EVs compared to EVs secreting from non-tumor adjacent tissues, which can be potentially used as cancer EV biomarkers or even to identify the cancer origin [202]. In another study, using chemical staining on lectins ABA and ACA, authors showed that O-glycans expression is not only different on normal vs tumor tissues but also on the EVs surface [35]. In the same study, they demonstrated that ABA- and ACA-positive EVs were significantly increased in the serum of 117 pancreatic patients compared to 98 normal controls with area under curve (AUC) values 0.838 and 0.810, respectively of the ROC curve. Though this study warrants validation with a larger cohort of clinical samples, these specific lectins (ABA and/or ACA) have the potential to be developed into a diagnostic test for the early detection of pancreatic cancer. Similarly, we have developed ITGA3-UEA assay where fucose binding lectin UEA can detect the aberrant fucosylations of ITGA3 + EVs which could facilitate the detection of bladder cancer [12]. These findings are definite examples of detection of EV-glycans with lectins and their potential in diagnostics.

Several cancer markers that are based on glycoproteins are already approved and routinely used; since these proteins are also found in EVs (Table 1), it stands to reason that altered EV glycosylation could emerge as a powerful tool for the diagnosis of cancer. Hence, this review has highlighted that lectin could serve as an effective tool for screening glycan-specific EV cancer biomarkers. Moreover, lectin-based approaches will be instrumental in developing EV-based therapeutics by advancing our understanding of EV glycobiology in disease.

EVs are heterogenous populations of nano-sized membrane vesicles that display glycans on their surface, some of which may be changed quantitatively and/or qualitatively during disease. Lectins can be used to recognize EV glycans and monitor disease-related changes, e.g.—in cancers. To realize the benefits of lectins, we recommend further research to assemble versatile panels of lectins to identify specific and sensitive EV-based biomarkers, especially for cancers.

### Supplementary Information

**Additional file 1: Supplementary Table 1.** Lectin used in EVs study and their major glycan specificities.

## Data Availability

Not applicable.

## Abbreviations

ADAM10: A disintegrin and metalloproteinase domain-containing protein 10
ADPKD: Autosomal dominant polycystic kidney disease
ADSC: Adipose-derived stem cells
CA 19-9: Carbohydrate antigen
CEA: Carcinoembryonic antigen
CD-MRP: Cation-dependent mannose 6-phosphate receptor
CI-MRP: Cation-independent mannose 6-phosphate receptor
CRDs: Carbohydrate-recognition domains
CTLDs: C-type lectin-like domains
DALDI-MS: Matrix-assisted laser desorption-mass spectrometry
EFF: Evanescent-field fluorescent
EIS-MS: Electrospray Ionization-mass spectrometry
EpCAM: Epithelium cell adhesion molecules
Eu^3+^-NP: Europium nanoparticle
EVs: Extracellular vesicles
FDA: Food and Drug Administration
GAG: Glycosaminoglycan
GalNAc: *N*-acetylgalactosamine
GBPs: Glycan-binding proteins
GC: Gas chromatography
GlcNAc: *N*-acetylglucosamine
GPI: Glycosylphosphatidylinositol
HCC: Hepatocellular carcinoma
hiPSCs: Human induced pluripotent stem cells
HPLC: High-performance liquid chromatography
IEC: Ion-exchange chromatography
IgSF: Immunoglobulin superfamily
ITGs: Integrins
JNPs: Janus nanoparticles
LC: Liquid chromatography
MS: Mass spectrometry
MUC: Mucins
TEM: Transmission electron microscopy
SCLC: Small-cell lung carcinoma
THP: Tamm-Horsefall protein

## References

[1] Fonseka P, Marzan AL, Mathivanan S (2021). Introduction to the community of extracellular vesicles. Subcell Biochem.

[2] Zaborowski MP, Balaj L, Breakefield XO, Lai CP (2015). Extracellular vesicles: composition, biological relevance, and methods of study. Bioscience.

[3] van Niel G, D'Angelo G, Raposo G (2018). Shedding light on the cell biology of extracellular vesicles. Nat Rev Mol Cell Biol.

[4] Huang D, Chen J, Hu D, Xie F, Yang T, Li Z (2021). Advances in biological function and clinical application of small extracellular vesicle membrane proteins. Front Oncol.

[5] Macedo-da-Silva J, Santiago VF, Rosa-Fernandes L, Marinho CRF, Palmisano G (2021). Protein glycosylation in extracellular vesicles: structural characterization and biological functions. Mol Immunol.

[6] Costa J (2017). Glycoconjugates from extracellular vesicles: Structures, functions and emerging potential as cancer biomarkers. Biochim Biophys Acta Rev Cancer.

[7] Ramazi S, Zahiri J (2021). Posttranslational modifications in proteins: resources, tools and prediction methods. Database (Oxford).

[8] Carnino JM, Ni K, Jin Y (2020). Post-translational modification regulates formation and cargo-Loading of extracellular vesicles. Front Immunol.

[9] Jo S, Qi Y, Im W (2016). Preferred conformations of N-glycan core pentasaccharide in solution and in glycoproteins. Glycobiology.

[10] Reynolds IS, Fichtner M, McNamara DA, Kay EW, Prehn JHM, Burke JP (2019). Mucin glycoproteins block apoptosis; promote invasion, proliferation, and migration; and cause chemoresistance through diverse pathways in epithelial cancers. Cancer Metastasis Rev.

[11] Prydz K (2015). Determinants of glycosaminoglycan (GAG) structure. Biomolecules.

[12] Islam MK, Dhondt B, Syed P, Khan M, Gidwani K, Webber J, et al. Integrins are enriched on aberrantly fucosylated tumour-derived urinary extracellular vesicles. JExBio;1:e64. 10.1002/jex2.64. 10.1002/jex2.64 PMC11080809 38939212

[13] Chen Z, Liang Q, Zeng H, Zhao Q, Guo Z, Zhong R (2020). Exosomal CA125 as a promising biomarker for ovarian cancer diagnosis. J Cancer.

[14] Zheng J, Hernandez JM, Doussot A, Bojmar L, Zambirinis CP, Costa-Silva B (2018). Extracellular matrix proteins and carcinoembryonic antigen-related cell adhesion molecules characterize pancreatic duct fluid exosomes in patients with pancreatic cancer. HPB (Oxford).

[15] Escrevente C, Grammel N, Kandzia S, Zeiser J, Tranfield EM, Conradt HS (2013). Sialoglycoproteins and N-glycans from secreted exosomes of ovarian carcinoma cells. PLoS One.

[16] Im H, Shao H, Park YI, Peterson VM, Castro CM, Weissleder R (2014). Label-free detection and molecular profiling of exosomes with a nano-plasmonic sensor. Nat Biotechnol.

[17] Runz S, Keller S, Rupp C, Stoeck A, Issa Y, Koensgen D (2007). Malignant ascites-derived exosomes of ovarian carcinoma patients contain CD24 and EpCAM. Gynecol Oncol.

[18] Sakaue T, Koga H, Iwamoto H, Nakamura T, Ikezono Y, Abe M (2019). Glycosylation of ascites-derived exosomal CD133: a potential prognostic biomarker in patients with advanced pancreatic cancer. Med Mol Morphol.

[19] Llorente A, Skotland T, Sylvänne T, Kauhanen D, Róg T, Orłowski A (2013). Molecular lipidomics of exosomes released by PC-3 prostate cancer cells. Biochim Biophys Acta.

[20] Christianson HC, Svensson KJ, van Kuppevelt TH, Li JP, Belting M (2013). Cancer cell exosomes depend on cell-surface heparan sulfate proteoglycans for their internalization and functional activity. Proc Natl Acad Sci U S A.

[21] Chen L, Brigstock DR (2016). Integrins and heparan sulfate proteoglycans on hepatic stellate cells (HSC) are novel receptors for HSC-derived exosomes. FEBS Lett.

[22] Friand V, David G, Zimmermann P (2015). Syntenin and syndecan in the biogenesis of exosomes. Biol Cell.

[23] Harada Y, Ohkawa Y, Maeda K, Kizuka Y, Taniguchi N (2021). Extracellular vesicles and glycosylation. Adv Exp Med Biol.

[24] Shimoda A, Miura R, Tateno H, Seo N, Shiku H, Sawada SI (2022). Assessment of surface glycan diversity on extracellular vesicles by lectin microarray and glycoengineering strategies for drug delivery applications. Small Methods.

[25] He J, Ren W, Wang W, Han W, Jiang L, Zhang D (2022). Exosomal targeting and its potential clinical application. Drug Deliv Transl Res.

[26] Kim H, Kim EH, Kwak G, Chi SG, Kim SH, Yang Y (2020). Exosomes: Cell-derived nanoplatforms for the delivery of cancer therapeutics. Int J Mol Sci.

[27] Cheng L, Hill AF (2022). Therapeutically harnessing extracellular vesicles. Nat Rev Drug Discov.

[28] Zhang Z, Cheng X, Jiang H, Gu J, Yin Y, Shen Z (2021). Quantitative proteomic analysis of glycosylated proteins enriched from urine samples with magnetic ConA nanoparticles identifies potential biomarkers for small cell lung cancer. J Pharm Biomed Anal.

[29] Escrevente C, Morais VA, Keller S, Soares CM, Altevogt P, Costa J (2008). Functional role of N-glycosylation from ADAM10 in processing, localization and activity of the enzyme. Biochim Biophys Acta.

[30] Yamamoto M, Harada Y, Suzuki T, Fukushige T, Yamakuchi M, Kanekura T (2019). Application of high-mannose-type glycan-specific lectin from Oscillatoria Agardhii for affinity isolation of tumor-derived extracellular vesicles. Anal Biochem.

[31] Huber V, Fais S, Iero M, Lugini L, Canese P, Squarcina P (2005). Human colorectal cancer cells induce T-cell death through release of proapoptotic microvesicles: role in immune escape. Gastroenterology.

[32] Lugini L, Valtieri M, Federici C, Cecchetti S, Meschini S, Condello M (2016). Exosomes from human colorectal cancer induce a tumor-like behavior in colonic mesenchymal stromal cells. Oncotarget.

[33] Staubach S, Schadewaldt P, Wendel U, Nohroudi K, Hanisch FG (2012). Differential glycomics of epithelial membrane glycoproteins from urinary exovesicles reveals shifts toward complex-type N-glycosylation in classical galactosemia. J Proteome Res.

[34] Batista BS, Eng WS, Pilobello KT, Hendricks-Muñoz KD, Mahal LK (2011). Identification of a conserved glycan signature for microvesicles. J Proteome Res.

[35] Yokose T, Kabe Y, Matsuda A, Kitago M, Matsuda S, Hirai M (2020). O-glycan-altered extracellular vesicles: a specific serum marker elevated in pancreatic cancer. Cancers (Basel).

[36] Chaiyawat P, Weeraphan C, Netsirisawan P, Chokchaichamnankit D, Srisomsap C, Svasti J (2016). Elevated O-GlcNAcylation of extracellular vesicle proteins derived from metastatic colorectal cancer cells. Cancer Genomics Proteomics.

[37] Freitas D, Balmaña M, Poças J, Campos D, Osório H, Konstantinidi A (2019). Different isolation approaches lead to diverse glycosylated extracellular vesicle populations. J Extracell Vesicles.

[38] Feng Y, Guo Y, Li Y, Tao J, Ding L, Wu J (2018). Lectin-mediated in situ rolling circle amplification on exosomes for probing cancer-related glycan pattern. Anal Chim Acta.

[39] Gomes J, Gomes-Alves P, Carvalho SB, Peixoto C, Alves PM, Altevogt P (2015). Extracellular vesicles from ovarian carcinoma cells display specific glycosignatures. Biomolecules.

[40] Netsirisawan P, Chokchaichamnankit D, Srisomsap C, Svasti J, Champattanachai V (2015). Proteomic analysis reveals aberrant O-GlcNAcylation of extracellular proteins from breast cancer cell secretion. Cancer Genomics Proteomics.

[41] Milutinović B, Goč S, Mitić N, Kosanović M, Janković M (2019). Surface glycans contribute to differences between seminal prostasomes from normozoospermic and oligozoospermic men. Ups J Med Sci.

[42] Murphy A, Cwiklinski K, Lalor R, O'Connell B, Robinson MW, Gerlach J (2020). Fasciola hepatica extracellular vesicles isolated from excretory-secretory products using a gravity flow method modulate dendritic cell phenotype and activity. PLoS Negl Trop Dis.

[43] Zhang H, Freitas D, Kim HS, Fabijanic K, Li Z, Chen H (2018). Identification of distinct nanoparticles and subsets of extracellular vesicles by asymmetric flow field-flow fractionation. Nat Cell Biol.

[44] Zou X, Yoshida M, Nagai-Okatani C, Iwaki J, Matsuda A, Tan B (2017). A standardized method for lectin microarray-based tissue glycome mapping. Sci Rep.

[45] Chen W, Wang R, Li D, Zuo C, Wen P, Liu H (2020). Comprehensive analysis of the glycome and glycoproteome of bovine milk-derived exosomes. J Agric Food Chem.

[46] Chen IH, Aguilar HA, Paez Paez JS, Wu X, Pan L, Wendt MK (2018). Analytical pipeline for discovery and verification of glycoproteins from plasma-derived extracellular vesicles as breast cancer biomarkers. Anal Chem.

[47] Harada Y, Nakajima K, Suzuki T, Fukushige T, Kondo K, Seino J (2020). Glycometabolic regulation of the biogenesis of small extracellular vesicles. Cell Rep.

[48] Martins ÁM, Ramos CC, Freitas D, Reis CA (2021). Glycosylation of cancer extracellular vesicles: capture strategies, functional roles and potential clinical applications. Cells.

[49] Williams C, Royo F, Aizpurua-Olaizola O, Pazos R, Boons GJ, Reichardt NC (2018). Glycosylation of extracellular vesicles: current knowledge, tools and clinical perspectives. J Extracell Vesicles.

[50] Costa J, Gatermann M, Nimtz M, Kandzia S, Glatzel M, Conradt HS (2018). N-glycosylation of extracellular vesicles from HEK-293 and glioma cell lines. Anal Chem.

[51] Echevarria J, Royo F, Pazos R, Salazar L, Falcon-Perez JM, Reichardt NC (2014). Microarray-based identification of lectins for the purification of human urinary extracellular vesicles directly from urine samples. ChemBioChem.

[52] Lin S, Zhou S, Yuan T (2020). The, "sugar-coated bullets" of cancer: Tumor-derived exosome surface glycosylation from basic knowledge to applications. Clin Transl Med.

[53] Surman M, Hoja-Łukowicz D, Szwed S, Drożdż A, Stępień E, Przybyło M (2018). Human melanoma-derived ectosomes are enriched with specific glycan epitopes. Life Sci.

[54] Krzyszczyk P, Acevedo A, Davidoff EJ, Timmins LM, Marrero-Berrios I, Patel M (2018). The growing role of precision and personalized medicine for cancer treatment. Technology (Singap World Sci).

[55] Yáñez-Mó M, Siljander PR, Andreu Z, Zavec AB, Borràs FE, Buzas EI (2015). Biological properties of extracellular vesicles and their physiological functions. J Extracell Vesicles.

[56] Liang LG, Kong MQ, Zhou S, Sheng YF, Wang P, Yu T (2017). An integrated double-filtration microfluidic device for isolation, enrichment and quantification of urinary extracellular vesicles for detection of bladder cancer. Sci Rep.

[57] Dhondt B, Van Deun J, Vermaerke S, de Marco A, Lumen N, De Wever O (2018). Urinary extracellular vesicle biomarkers in urological cancers: From discovery towards clinical implementation. Int J Biochem Cell Biol.

[58] Melo SA, Luecke LB, Kahlert C, Fernandez AF, Gammon ST, Kaye J (2015). Glypican-1 identifies cancer exosomes and detects early pancreatic cancer. Nature.

[59] Vermassen T, D'Herde K, Jacobus D, Van Praet C, Poelaert F, Lumen N (2017). Release of urinary extracellular vesicles in prostate cancer is associated with altered urinary N-glycosylation profile. J Clin Pathol.

[60] Nyalwidhe JO, Betesh LR, Powers TW, Jones EE, White KY, Burch TC (2013). Increased bisecting N-acetylglucosamine and decreased branched chain glycans of N-linked glycoproteins in expressed prostatic secretions associated with prostate cancer progression. Proteomics Clin Appl.

[61] Li Y, Zhang Y, Qiu F, Qiu Z (2011). Proteomic identification of exosomal LRG1: a potential urinary biomarker for detecting NSCLC. Electrophoresis.

[62] Niu L, Song X, Wang N, Xue L, Xie L (2019). Tumor-derived exosomal proteins as diagnostic biomarkers in non-small cell lung cancer. Cancer Sci.

[63] Pan D, Chen J, Feng C, Wu W, Wang Y, Tong J (2019). Preferential localization of MUC1 glycoprotein in exosomes secreted by non-small cell lung carcinoma cells. Int J Mol Sci.

[64] Kharaziha P, Chioureas D, Rutishauser D, Baltatzis G, Lennartsson L, Fonseca P (2015). Molecular profiling of prostate cancer derived exosomes may reveal a predictive signature for response to docetaxel. Oncotarget.

[65] Lazar I, Clement E, Ducoux-Petit M, Denat L, Soldan V, Dauvillier S (2015). Proteome characterization of melanoma exosomes reveals a specific signature for metastatic cell lines. Pigment Cell Melanoma Res.

[66] He M, Qin H, Poon TC, Sze SC, Ding X, Co NN (2015). Hepatocellular carcinoma-derived exosomes promote motility of immortalized hepatocyte through transfer of oncogenic proteins and RNAs. Carcinogenesis.

[67] Welton JL, Khanna S, Giles PJ, Brennan P, Brewis IA, Staffurth J (2010). Proteomics analysis of bladder cancer exosomes. Mol Cell Proteomics.

[68] Admyre C, Johansson SM, Qazi KR, Filén JJ, Lahesmaa R, Norman M (2007). Exosomes with immune modulatory features are present in human breast milk. J Immunol.

[69] Staubach S, Razawi H, Hanisch FG (2009). Proteomics of MUC1-containing lipid rafts from plasma membranes and exosomes of human breast carcinoma cells MCF-7. Proteomics.

[70] Yu S, Li Y, Liao Z, Wang Z, Qian L, Zhao J (2020). Plasma extracellular vesicle long RNA profiling identifies a diagnostic signature for the detection of pancreatic ductal adenocarcinoma. Gut.

[71] Moravec R, Divi R, Verma M (2017). Detecting circulating tumor material and digital pathology imaging during pancreatic cancer progression. World J Gastrointest Oncol.

[72] Lane JS, Hoff DV, Cridebring D, Goel A (2020). Extracellular vesicles in diagnosis and treatment of pancreatic cancer: current State and future perspectives. Cancers (Basel).

[73] Choi DS, Park JO, Jang SC, Yoon YJ, Jung JW, Choi DY (2011). Proteomic analysis of microvesicles derived from human colorectal cancer ascites. Proteomics.

[74] Silvers CR, Miyamoto H, Messing EM, Netto GJ, Lee YF (2017). Characterization of urinary extracellular vesicle proteins in muscle-invasive bladder cancer. Oncotarget.

[75] van Herwijnen MJ, Zonneveld MI, Goerdayal S, Nolte-'t Hoen EN, Garssen J, Stahl B (2016). Comprehensive proteomic analysis of human milk-derived extracellular vesicles unveils a novel functional proteome distinct from other milk components. Mol Cell Proteomics.

[76] Liu X, Chinello C, Musante L, Cazzaniga M, Tataruch D, Calzaferri G (2015). Intraluminal proteome and peptidome of human urinary extracellular vesicles. Proteomics Clin Appl.

[77] Hurwitz SN, Rider MA, Bundy JL, Liu X, Singh RK, Meckes DG (2016). Proteomic profiling of NCI-60 extracellular vesicles uncovers common protein cargo and cancer type-specific biomarkers. Oncotarget.

[78] Diamant M, Nieuwland R, Pablo RF, Sturk A, Smit JW, Radder JK (2002). Elevated numbers of tissue-factor exposing microparticles correlate with components of the metabolic syndrome in uncomplicated type 2 diabetes mellitus. Circulation.

[79] Wubbolts R, Leckie RS, Veenhuizen PT, Schwarzmann G, Möbius W, Hoernschemeyer J (2003). Proteomic and biochemical analyses of human B cell-derived exosomes. Potential implications for their function and multivesicular body formation. J Biol Chem.

[80] Fraser KB, Moehle MS, Daher JP, Webber PJ, Williams JY, Stewart CA (2013). LRRK2 secretion in exosomes is regulated by 14-3-3. Hum Mol Genet.

[81] Gonzales PA, Pisitkun T, Hoffert JD, Tchapyjnikov D, Star RA, Kleta R (2009). Large-scale proteomics and phosphoproteomics of urinary exosomes. J Am Soc Nephrol.

[82] Bruschi M, Santucci L, Ravera S, Bartolucci M, Petretto A, Calzia D (2018). Metabolic signature of microvesicles from umbilical cord mesenchymal stem cells of preterm and term infants. Proteomics Clin Appl.

[83] Hong BS, Cho JH, Kim H, Choi EJ, Rho S, Kim J (2009). Colorectal cancer cell-derived microvesicles are enriched in cell cycle-related mRNAs that promote proliferation of endothelial cells. BMC Genomics.

[84] Sinha A, Ignatchenko V, Ignatchenko A, Mejia-Guerrero S, Kislinger T (2014). In-depth proteomic analyses of ovarian cancer cell line exosomes reveals differential enrichment of functional categories compared to the NCI 60 proteome. Biochem Biophys Res Commun.

[85] Principe S, Jones EE, Kim Y, Sinha A, Nyalwidhe JO, Brooks J (2013). In-depth proteomic analyses of exosomes isolated from expressed prostatic secretions in urine. Proteomics.

[86] Musante L, Saraswat M, Duriez E, Byrne B, Ravidà A, Domon B (2012). Biochemical and physical characterisation of urinary nanovesicles following CHAPS treatment. PLoS One.

[87] Utleg AG, Yi EC, Xie T, Shannon P, White JT, Goodlett DR (2003). Proteomic analysis of human prostasomes. Prostate.

[88] Oeyen E, Van Mol K, Baggerman G, Willems H, Boonen K, Rolfo C (2018). Ultrafiltration and size exclusion chromatography combined with asymmetrical-flow field-flow fractionation for the isolation and characterisation of extracellular vesicles from urine. J Extracell Vesicles.

[89] Bruno S, Grange C, Deregibus MC, Calogero RA, Saviozzi S, Collino F (2009). Mesenchymal stem cell-derived microvesicles protect against acute tubular injury. J Am Soc Nephrol.

[90] Zubiri I, Posada-Ayala M, Sanz-Maroto A, Calvo E, Martin-Lorenzo M, Gonzalez-Calero L (2014). Diabetic nephropathy induces changes in the proteome of human urinary exosomes as revealed by label-free comparative analysis. J Proteomics.

[91] Manek R, Moghieb A, Yang Z, Kumar D, Kobessiy F, Sarkis GA (2018). Protein biomarkers and neuroproteomics characterization of microvesicles/exosomes from human cerebrospinal fluid following traumatic brain injury. Mol Neurobiol.

[92] Perez-Hernandez D, Gutiérrez-Vázquez C, Jorge I, López-Martín S, Ursa A, Sánchez-Madrid F (2013). The intracellular interactome of tetraspanin-enriched microdomains reveals their function as sorting machineries toward exosomes. J Biol Chem.

[93] Taylor ME, Drickamer K, Schnaar RL, Etzler ME, Varki A. Discovery and classification of glycan-binding proteins. 2017. In: Varki A, Cummings RD, Esko JD, Stanley P, Hart GW, Aebi M, Darvill AG, Kinoshita T, Packer NH, Prestegard JH, Schnaar RL, Seeberger PH, editors. Essentials of glycobiology. 3rd ed. Cold Spring Harbor (NY): Cold Spring Harbor Laboratory Press; 2015–2017. Chapter 28.

[94] Sharon N, Lis H (2004). History of lectins: from hemagglutinins to biological recognition molecules. Glycobiology.

[95] Boyd WC, Shapleigh E (1954). Antigenic relations of blood group antigens as suggested by tests with lectins. J Immunol.

[96] Boyd WC, Shapleigh E (1954). Specific precipitating activity of plant agglutinins (lectins). Science.

[97] Kilpatrick DC (2002). Animal lectins: a historical introduction and overview. Biochim Biophys Acta.

[98] Sharon N (1987). Bacterial lectins, cell-cell recognition and infectious disease. FEBS Lett.

[99] Kobayashi Y, Kawagishi H (2014). Fungal lectins: a growing family. Methods Mol Biol.

[100] Varki A, Cummings RD, Esko JD, Stanley P, Hart GW, Aebi M, Darvill AG, Kinoshita T, Packer NH, Prestegard JH, Schnaar RL, Seeberger PH, editors. Essentials of glycobiology. 3rd ed. Cold Spring Harbor (NY): Cold Spring Harbor Laboratory Press; 2015–2017. 27010055

[101] Rutishauser U, Sachs L (1975). Cell-to-cell binding induced by different lectins. J Cell Biol.

[102] Brudner M, Karpel M, Lear C, Chen L, Yantosca LM, Scully C (2013). Lectin-dependent enhancement of Ebola virus infection via soluble and transmembrane C-type lectin receptors. PLoS One.

[103] Gabius HJ (1997). Animal lectins. Eur J Biochem.

[104] Mishra A, Behura A, Mawatwal S, Kumar A, Naik L, Mohanty SS (2019). Structure-function and application of plant lectins in disease biology and immunity. Food Chem Toxicol.

[105] Hashim OH, Jayapalan JJ, Lee CS (2017). Lectins: an effective tool for screening of potential cancer biomarkers. PeerJ.

[106] Lis H, Sharon N (1986). Lectins as molecules and as tools. Annu Rev Biochem.

[107] Varki A, Schnaar RL, Crocker PR. I-Type Lectins. 2017. In: Varki A, Cummings RD, Esko JD, Stanley P, Hart GW, Aebi M, Darvill AG, Kinoshita T, Packer NH, Prestegard JH, Schnaar RL, Seeberger PH, editors. Essentials of glycobiology. 3rd ed. Cold Spring Harbor (NY): Cold Spring Harbor Laboratory Press; 2015–2017. Chapter 35.

[108] Pathan M, Fonseka P, Chitti SV, Kang T, Sanwlani R, Van Deun J (2019). Vesiclepedia 2019: a compendium of RNA, proteins, lipids and metabolites in extracellular vesicles. Nucleic Acids Res.

[109] Keerthikumar S, Chisanga D, Ariyaratne D, Al Saffar H, Anand S, Zhao K (2016). ExoCarta: a web-based compendium of exosomal cargo. J Mol Biol.

[110] Gabius HJ, Engelhardt R, Cramer F (1985). Endogenous tumor lectins: a new class of tumor markers and targets for therapy?. Med Hypotheses.

[111] Popa SJ, Stewart SE, Moreau K (2018). Unconventional secretion of annexins and galectins. Semin Cell Dev Biol.

[112] Barrès C, Blanc L, Bette-Bobillo P, André S, Mamoun R, Gabius HJ (2010). Galectin-5 is bound onto the surface of rat reticulocyte exosomes and modulates vesicle uptake by macrophages. Blood.

[113] Gerlach JQ, Maguire CM, Krüger A, Joshi L, Prina-Mello A, Griffin MD (2017). Urinary nanovesicles captured by lectins or antibodies demonstrate variations in size and surface glycosylation profile. Nanomedicine (Lond).

[114] Escrevente C, Keller S, Altevogt P, Costa J (2011). Interaction and uptake of exosomes by ovarian cancer cells. BMC Cancer.

[115] Samsonov R, Shtam T, Burdakov V, Glotov A, Tsyrlina E, Berstein L (2016). Lectin-induced agglutination method of urinary exosomes isolation followed by mi-RNA analysis: application for prostate cancer diagnostic. Prostate.

[116] Williams C, Pazos R, Royo F, González E, Roura-Ferrer M, Martinez A (2019). Assessing the role of surface glycans of extracellular vesicles on cellular uptake. Sci Rep.

[117] Gerlach JQ, Krüger A, Gallogly S, Hanley SA, Hogan MC, Ward CJ (2013). Surface glycosylation profiles of urine extracellular vesicles. PLoS One.

[118] Krishnamoorthy L, Bess JW, Preston AB, Nagashima K, Mahal LK (2009). HIV-1 and microvesicles from T cells share a common glycome, arguing for a common origin. Nat Chem Biol.

[119] Shtam TA, Burdakov VS, Landa SB, Naryzhny SN, Bairamukov VY, Malek AV (2017). Aggregation by lectin-methodical approach for effective isolation of exosomes from cell culture supernatant for proteome profiling. Tsitologiia.

[120] Wang S, Cesca F, Loers G, Schweizer M, Buck F, Benfenati F (2011). Synapsin I is an oligomannose-carrying glycoprotein, acts as an oligomannose-binding lectin, and promotes neurite outgrowth and neuronal survival when released via glia-derived exosomes. J Neurosci.

[121] Shimoda A, Tahara Y, Sawada SI, Sasaki Y, Akiyoshi K (2017). Glycan profiling analysis using evanescent-field fluorescence-assisted lectin array: importance of sugar recognition for cellular uptake of exosomes from mesenchymal stem cells. Biochem Biophys Res Commun.

[122] Shimoda A, Sawada SI, Sasaki Y, Akiyoshi K (2019). Exosome surface glycans reflect osteogenic differentiation of mesenchymal stem cells: profiling by an evanescent field fluorescence-assisted lectin array system. Sci Rep.

[123] Harada Y, Suzuki T, Fukushige T, Kizuka Y, Yagi H, Yamamoto M (2019). Generation of the heterogeneity of extracellular vesicles by membrane organization and sorting machineries. Biochim Biophys Acta Gen Subj.

[124] Desantis S, Accogli G, Albrizio M, Rossi R, Cremonesi F, Lange CA (2019). Glycan profiling analysis of equine amniotic progenitor mesenchymal cells and their derived extracellular microvesicles. Stem Cells Dev.

[125] de la Torre-Escudero E, Gerlach JQ, Bennett APS, Cwiklinski K, Jewhurst HL, Huson KM (2019). Surface molecules of extracellular vesicles secreted by the helminth pathogen Fasciola hepatica direct their internalisation by host cells. PLoS Negl Trop Dis.

[126] Saito S, Hiemori K, Kiyoi K, Tateno H (2018). Glycome analysis of extracellular vesicles derived from human induced pluripotent stem cells using lectin microarray. Sci Rep.

[127] Nishida-Aoki N, Tominaga N, Kosaka N, Ochiya T (2020). Altered biodistribution of deglycosylated extracellular vesicles through enhanced cellular uptake. J Extracell Vesicles.

[128] Tan Z, Cao L, Wu Y, Wang B, Song Z, Yang J (2020). Bisecting GlcNAc modification diminishes the pro-metastatic functions of small extracellular vesicles from breast cancer cells. J Extracell Vesicles.

[129] Kosanovic M, Jankovic M (2014). Isolation of urinary extracellular vesicles from tamm- horsfall protein-depleted urine and their application in the development of a lectin-exosome-binding assay. Biotechniques.

[130] Choi Y, Park U, Koo HJ, Park JS, Lee DH, Kim K (2021). Exosome-mediated diagnosis of pancreatic cancer using lectin-conjugated nanoparticles bound to selective glycans. Biosens Bioelectron.

[131] Cummings RD, L. Schnaar R. R-Type Lectins. 2017. In: Varki A, Cummings RD, Esko JD, Stanley P, Hart GW, Aebi M, Darvill AG, Kinoshita T, Packer NH, Prestegard JH, Schnaar RL, Seeberger PH, editors. Essentials of glycobiology. 3rd ed. Cold Spring Harbor (NY): Cold Spring Harbor Laboratory Press; 2015–2017. Chapter 31.

[132] McCourt PA, Ek B, Forsberg N, Gustafson S (1994). Intercellular adhesion molecule-1 is a cell surface receptor for hyaluronan. J Biol Chem.

[133] Kiriyama K, Itoh K (2020). Glycan recognition and application of P-type lectins. Methods Mol Biol.

[134] Dahms N, Hancock MK (2002). P-type lectins. Biochim Biophys Acta.

[135] Brown GD, Willment JA, Whitehead L (2018). C-type lectins in immunity and homeostasis. Nat Rev Immunol.

[136] Islam MK, Syed P, Lehtinen L, Leivo J, Gidwani K, Wittfooth S (2019). A nanoparticle-based approach for the detection of extracellular vesicles. Sci Rep.

[137] Kuipers ME, Nolte-'t Hoen ENM, van der Ham AJ, Ozir-Fazalalikhan A, Nguyen DL, de Korne CM (2020). DC-SIGN mediated internalisation of glycosylated extracellular vesicles from Schistosoma mansoni increases activation of monocyte-derived dendritic cells. J Extracell Vesicles.

[138] Sung PS, Hsieh SL (2021). C-type lectins and extracellular vesicles in virus-induced NETosis. J Biomed Sci.

[139] Słomka A, Urban SK, Lukacs-Kornek V, Żekanowska E, Kornek M (2018). Large extracellular vesicles: have we found the holy grail of inflammation?. Front Immunol.

[140] Ivetic A, Hoskins Green HL, Hart SJ (2019). L-selectin: A major regulator of leukocyte adhesion, migration and signaling. Front Immunol.

[141] McEver RP (2015). Selectins: initiators of leucocyte adhesion and signalling at the vascular wall. Cardiovasc Res.

[142] Yoshida Y (2003). A novel role for N-glycans in the ERAD system. J Biochem.

[143] Glenn KA, Nelson RF, Wen HM, Mallinger AJ, Paulson HL (2008). Diversity in tissue expression, substrate binding, and SCF complex formation for a lectin family of ubiquitin ligases. J Biol Chem.

[144] Lenza MP, Atxabal U, Oyenarte I, Jiménez-Barbero J, Ereño-Orbea J (2020). Current status on therapeutic molecules targeting siglec receptors. Cells.

[145] Rodrigues JG, Balmaña M, Macedo JA, Poças J, Fernandes Â, de Freitas-Junior JCM (2018). Glycosylation in cancer: Selected roles in tumour progression, immune modulation and metastasis. Cell Immunol.

[146] Saunderson SC, Dunn AC, Crocker PR, McLellan AD (2014). CD169 mediates the capture of exosomes in spleen and lymph node. Blood.

[147] Dusoswa SA, Horrevorts SK, Ambrosini M, Kalay H, Paauw NJ, Nieuwland R (2019). Glycan modification of glioblastoma-derived extracellular vesicles enhances receptor-mediated targeting of dendritic cells. J Extracell Vesicles.

[148] Li Y, Zhou J, Zhuo Q, Zhang J, Xie J, Han S (2019). Malignant ascite-derived extracellular vesicles inhibit T cell activity by upregulating Siglec-10 expression. Cancer Manag Res.

[149] Takasaki N, Tachibana K, Ogasawara S, Matsuzaki H, Hagiuda J, Ishikawa H (2014). A heterozygous mutation of GALNTL5 affects male infertility with impairment of sperm motility. Proc Natl Acad Sci U S A.

[150] Thijssen VL, Heusschen R, Caers J, Griffioen AW (2015). Galectin expression in cancer diagnosis and prognosis: a systematic review. Biochim Biophys Acta.

[151] Girotti MR, Salatino M, Dalotto-Moreno T, Rabinovich GA (2020). Sweetening the hallmarks of cancer: Galectins as multifunctional mediators of tumor progression. J Exp Med.

[152] Maybruck BT, Pfannenstiel LW, Diaz-Montero M, Gastman BR (2017). Tumor-derived exosomes induce CD8(+) T cell suppressors. J Immunother Cancer.

[153] Liang B, Peng P, Chen S, Li L, Zhang M, Cao D (2013). Characterization and proteomic analysis of ovarian cancer-derived exosomes. J Proteomics.

[154] Mathivanan S, Lim JW, Tauro BJ, Ji H, Moritz RL, Simpson RJ (2010). Proteomics analysis of A33 immunoaffinity-purified exosomes released from the human colon tumor cell line LIM1215 reveals a tissue-specific protein signature. Mol Cell Proteomics.

[155] Jones JL, Saraswati S, Block AS, Lichti CF, Mahadevan M, Diekman AB (2010). Galectin-3 is associated with prostasomes in human semen. Glycoconj J.

[156] Bänfer S, Schneider D, Dewes J, Strauss MT, Freibert SA, Heimerl T (2018). Molecular mechanism to recruit galectin-3 into multivesicular bodies for polarized exosomal secretion. Proc Natl Acad Sci U S A.

[157] Díaz-Alvarez L, Ortega E (2017). The many roles of galectin-3, a multifaceted molecule, in innate immune responses against pathogens. Mediators Inflamm.

[158] Almeida F, Wolf JM, da Silva TA, DeLeon-Rodriguez CM, Rezende CP, Pessoni AM (2017). Galectin-3 impacts cryptococcus neoformans infection through direct antifungal effects. Nat Commun.

[159] Hatanaka O, Rezende CP, Moreno P, Freitas Fernandes F, Oliveira Brito PKM, Martinez R, Coelho C, Roque-Barreira MC, Casadevall A, Almeida F (2019). Galectin-3 inhibits paracoccidioides brasiliensis growth and impacts paracoccidioidomycosis through multiple mechanisms. mSphere.

[160] Keryer-Bibens C, Pioche-Durieu C, Villemant C, Souquère S, Nishi N, Hirashima M (2006). Exosomes released by EBV-infected nasopharyngeal carcinoma cells convey the viral latent membrane protein 1 and the immunomodulatory protein galectin 9. BMC Cancer.

[161] Liang Y, Eng WS, Colquhoun DR, Dinglasan RR, Graham DR, Mahal LK (2014). Complex N-linked glycans serve as a determinant for exosome/microvesicle cargo recruitment. J Biol Chem.

[162] Stowell SR, Ju T, Cummings RD (2015). Protein glycosylation in cancer. Annu Rev Pathol.

[163] Wang M, Zhu J, Lubman DM, Gao C (2019). Aberrant glycosylation and cancer biomarker discovery: a promising and thorny journey. Clin Chem Lab Med.

[164] Kailemia MJ, Park D, Lebrilla CB (2017). Glycans and glycoproteins as specific biomarkers for cancer. Anal Bioanal Chem.

[165] Badr HA, Alsadek DM, Darwish AA, Elsayed AI, Bekmanov BO, Khussainova EM (2014). Lectin approaches for glycoproteomics in FDA-approved cancer biomarkers. Expert Rev Proteomics.

[166] Durand G, Seta N (2000). Protein glycosylation and diseases: blood and urinary oligosaccharides as markers for diagnosis and therapeutic monitoring. Clin Chem.

[167] Kirwan A, Utratna M, O'Dwyer ME, Joshi L, Kilcoyne M (2015). Glycosylation-based serum biomarkers for cancer diagnostics and prognostics. Biomed Res Int.

[168] Khien VV, Mao HV, Chinh TT, Ha PT, Bang MH, Lac BV (2001). Clinical evaluation of lentil lectin-reactive alpha-fetoprotein-L3 in histology-proven hepatocellular carcinoma. Int J Biol Markers.

[169] Kuno A, Uchiyama N, Koseki-Kuno S, Ebe Y, Takashima S, Yamada M (2005). Evanescent-field fluorescence-assisted lectin microarray: a new strategy for glycan profiling. Nat Methods.

[170] Harada Y, Kizuka Y, Tokoro Y, Kondo K, Yagi H, Kato K (2019). N-glycome inheritance from cells to extracellular vesicles in B16 melanomas. FEBS Lett.

[171] Vinod R, Mahran R, Routila E, Leivo J, Pettersson K, Gidwani K (2021). Nanoparticle-aided detection of colorectal cancer-associated glycoconjugates of extracellular vesicles in human serum. Int J Mol Sci.

[172] Terävä J, Verhassel A, Botti O, Islam MK, Leivo J, Wittfooth S (2021). Primary breast cancer biomarkers based on glycosylation and extracellular vesicles detected from human serum. Cancer Rep (Hoboken).

[173] Hu S, Wong DT (2009). Lectin microarray. Proteomics Clin Appl.

[174] Bertokova A, Svecova N, Kozics K, Gabelova A, Vikartovska A, Jane E (2022). Exosomes from prostate cancer cell lines: isolation optimisation and characterisation. Biomed Pharmacother.

[175] Hayashi Y, Yimiti D, Sanada Y, Ding C, Omoto T, Ogura T (2022). The therapeutic capacity of bone marrow MSC-derived extracellular vesicles in Achilles tendon healing is passage-dependent and indicated by specific glycans. FEBS Lett.

[176] Clos-Sansalvador M, Garcia SG, Morón-Font M, Williams C, Reichardt NC, Falcón-Pérez JM (2022). N-glycans in immortalized mesenchymal stromal cell-derived extracellular vesicles are critical for EV-cell interaction and functional activation of endothelial cells. Int J Mol Sci.

[177] Musante L, Tataruch-Weinert D, Kerjaschki D, Henry M, Meleady P, Holthofer H (2017). Residual urinary extracellular vesicles in ultracentrifugation supernatants after hydrostatic filtration dialysis enrichment. J Extracell Vesicles.

[178] Wang D, Sun W (2014). Urinary extracellular microvesicles: isolation methods and prospects for urinary proteome. Proteomics.

[179] Boriachek K, Islam MN, Möller A, Salomon C, Nguyen NT, Hossain MSA, Yamauchi Y, Shiddiky MJA. Biological functions and current advances in isolation and detection strategies for exosome nanovesicles. Small. 2018;14(6). 10.1002/smll.201702153 29282861

[180] Benecke L, Chiang DM, Ebnoether E, Pfaffl MW, Muller L (2022). Isolation and analysis of tumor-derived extracellular vesicles from head and neck squamous cell carcinoma plasma by galectin-based glycan recognition particles. Int J Oncol.

[181] Kanao E, Wada S, Nishida H, Kubo T, Tanigawa T, Imami K (2022). Classification of extracellular vesicles based on surface glycan structures by spongy-like separation media. Anal Chem.

[182] Nagae M, Soga K, Morita-Matsumoto K, Hanashima S, Ikeda A, Yamamoto K (2014). Phytohemagglutinin from Phaseolus vulgaris (PHA-E) displays a novel glycan recognition mode using a common legume lectin fold. Glycobiology.

[183] Kondo K, Harada Y, Nakano M, Suzuki T, Fukushige T, Hanzawa K (2022). Identification of distinct N-glycosylation patterns on extracellular vesicles from small-cell and non-small-cell lung cancer cells. J Biol Chem.

[184] Surman M, Wilczak M, Przybyło M (2022). Lectin-based study reveals the presence of disease-relevant glycoepitopes in bladder cancer cells and ectosomes. Int J Mol Sci.

[185] Zhang J, Qin Y, Jiang Q, Li F, Jing X, Cao L (2022). Glycopattern alteration of glycoproteins in gastrointestinal cancer cell lines and their cell-derived exosomes. J Proteome Res.

[186] Gidwani K, Huhtinen K, Kekki H, van Vliet S, Hynninen J, Koivuviita N (2016). A nanoparticle-lectin immunoassay improves discrimination of serum ca125 from malignant and benign sources. Clin Chem.

[187] Härmä H, Soukka T, Lövgren T (2001). Europium nanoparticles and time-resolved fluorescence for ultrasensitive detection of prostate-specific antigen. Clin Chem.

[188] Syed P, Gidwani K, Kekki H, Leivo J, Pettersson K, Lamminmaki U (2016). Role of lectin microarrays in cancer diagnosis. Proteomics.

[189] Islam MK, Syed P, Dhondt B, Gidwani K, Pettersson K, Lamminmäki U (2021). Detection of bladder cancer with aberrantly fucosylated ITGA3. Anal Biochem.

[190] Dong L, Zieren RC, Wang Y, de Reijke TM, Xue W, Pienta KJ (2019). Recent advances in extracellular vesicle research for urological cancers: from technology to application. Biochim Biophys Acta Rev Cancer.

[191] Srivastava A, Amreddy N, Pareek V, Chinnappan M, Ahmed R, Mehta M (2020). Progress in extracellular vesicle biology and their application in cancer medicine. Wiley Interdiscip Rev Nanomed Nanobiotechnol.

[192] McKiernan J, Donovan MJ, O'Neill V, Bentink S, Noerholm M, Belzer S (2016). A novel urine exosome gene expression assay to predict high-grade prostate cancer at initial biopsy. JAMA Oncol.

[193] Pinsky PF, Zhu CS (2011). Building multi-marker algorithms for disease prediction-the role of correlations among markers. Biomark Insights.

[194] Mattox DE, Bailey-Kellogg C (2021). Comprehensive analysis of lectin-glycan interactions reveals determinants of lectin specificity. PLoS Comput Biol.

[195] Choi HK, Lee D, Singla A, Kwon JS, Wu HJ (2019). The influence of heteromultivalency on lectin-glycan binding behavior. Glycobiology.

[196] Haab BB (2012). Using lectins in biomarker research: addressing the limitations of sensitivity and availability. Proteomics Clin Appl.

[197] Varki A, Cummings RD, Esko JD, Stanley P, Hart GW, Aebi M, Mohnen D, Kinoshita T, Packer NH, Prestegard JH, Schnaar RL, Seeberger PH, editors. Essentials of glycobiology. 4th ed. Cold Spring Harbor (NY): Cold Spring Harbor Laboratory Press; 2022. 35536922

[198] Carrillo C, Cordoba-Diaz D, Cordoba-Diaz M, Girbés T, Jiménez P (2017). Effects of temperature, pH and sugar binding on the structures of lectins ebulin f and SELfd. Food Chem.

[199] Chettri D, Boro M, Sarkar L, Verma AK (2021). Lectins: biological significance to biotechnological application. Carbohydr Res.

[200] Van Damme EJM (2022). 35 years in plant lectin research: a journey from basic science to applications in agriculture and medicine. Glycoconj J.

[201] Walker SA, Aguilar Díaz De León JS, Busatto S, Wurtz GA, Zubair AC, Borges CR (2020). Glycan node analysis of plasma-derived extracellular vesicles. Cells.

[202] Hoshino A, Kim HS, Bojmar L, Gyan KE, Cioffi M, Hernandez J (2020). Extracellular vesicle and particle biomarkers define multiple human cancers. Cell.

