# Supplementary material for: Lectins as potential tools for cancer biomarker discovery from extracellular vesicles
Source: Biomark Res. 2023 Sep 29;11:85. doi: 10.1186/s40364-023-00520-6 (PMC10540341; doi:10.1186/s40364-023-00520-6)
Supplement: Supplementary file 1 — Additional file 1: Supplementary Table 1. Lectin used in EVs study and their major glycan specificities. [file 40364_2023_520_MOESM1_ESM.docx]

## Supplementary Table 1: Lectin used in EVs study and their major glycan specificities.

| **Lectin** | **Full name of lectin/agglutinin** | **Major carbohydrate binding specificities** |
| --- | --- | --- |
| AAL | *Aleuria aurantia* | α1-6Fuc |
| ABL | *Agaricus bisporus* | Gal, Galβ1-3 GalNac |
| ACA | *Amaranthus caudatus* | Galβ1-3GalNAc(T-antigen), Siaα2-3Galβ1-3GalNAc (sialyl T) |
| ACG | *Agrocybe cylindracea* | Galβ1-3Gal, Siaα2-3Galβ1-4GlcNAc |
| AIA | *Artocarpus intergrifolia* (Jacalin) | GlcNAcβ1-3GalNAc (Core3), sialyl T, T-antigen, and Tn-antigen |
| AMA | *Arum maculatum* | Biantennary core (GlcNAc), high mannose,  LacNAc |
| AOL | *Aspergillus oryzae* | Fucα1,2Gal and Fucα1-6 |
| ASA | *Allium sativa* | High Man (Man9-GlcNAc2) |
| BPL | *Bauhinia purpurea* | Galβ1-3GalNAc |
| CAL | *Caragana arborescens* | Gal or GalNAc |
| Calsepa | *Calystegia sepium* | α‐Man, α‐Glc |
| CCA | *Cancer antennarius* | 9-O-Acetyl NeuAc and 4-O-Acetyl  NeuAc |
| Con A | *Canavalia ensiformis* | Branched and terminal mannose,  terminal GlcNAc |
| CPA | *Cicer arietinum* | Complex N-glycans |
| CVN | Cyanovirin | High Man |
| DBA | *Dolichos biflorus* | α- and β-GalNAc |
| DC SIGN | Dendritic Cell-Specific Intercellular adhesion molecule-3-Grabbing Non-integrin (DC-SIGN) | Non-sialylated Lewis antigens and high mannose-type structures |
| Dectin-1 | C-type lectin | Beta-glucans |
| Dectin-2 | C-type lectin | High mannose |
| DSL | *Datura stramonium* | (β-1,4) linked *N*-acetylglucosamine oligomers |
| ECA | *Erythrina cristagalli* | GalNAcβ1-4GlcNAc, Galβ1-4GlcNAc |
| EEA | *Euonymus eurpaeus* | Galα1-3(Fucα1-2)Gal |
| Ficolin-1 | Oligomeric lectin-1 | GlcNAc, GalNAc; sialic acid |
| Ficolin-2 | Oligomeric lectin-2 | GlcNAc (acetyl group), -(1]3)-D-glucan, *N*-acetylneuraminic acid |
| Ficolin-3 | Oligomeric lectin-3 | GlcNAc, GalNAc, fucose |
| Gal-1 | Galectin-1 | Galβ1-3/4GlcNAc |
| Gal-3 | Galectin-3 | Galactomannans, mannan |
| Gal-4 | Galectin-4 | SO_3_->3Galβ1->3GalNAc pyranoside |
| Gal-9 | Galectin-9 | GalNacα1-3GalNacβ1-3Galα1- 4Galβ1-4Glc |
| GNA | *Galanthus nivalis* | Terminal Man α1-3 |
| GNL | *Galanthus nivalis* | (α-1,3) mannose |
| GRFT | Griffithsin | High Man |
| *GSL-II* | *Griffonia simplicifolia-II* | GlcNAc |
| GSL-I A4 | *Griffonia simplicifolia-I A4* | αGal, GalNAc |
| GSL-I B4 | *Griffonia simplicifolia-I B4* | αGal, GalNAc |
| HAA | *Helix aspersa* | Terminal GalNAc |
| PHA-M | *Phaseolus vulgaris-*M | GalNAc |
| HHL/AL | *Hippeastrum hybrid /Amarylis Lectin* | Manα1-3 and 1-6 |
| HPA | *Helix pomatia* | α-GalNAc |
| IRA | *lris hybrid* | GalNAc |
| LBA | *Phaseolus lunatus* | GalNAcα1-3(Fucα1-2)Gal |
| LCA/LCH | *Lens culinaris* | Complex and high mannose, Fuc α1-6 |
| LEL/TL | *Lypersicon esculentum* (Tomato)/ *Tulipa sp.* | Poly-LacNAc, β1-4 GlcNAc oligomers |
| LFA | *Limax flavus* | α -NeuAc (O-glycans) |
| LPA | *Limulus polphemus* | α -NeuAc |
| LTL | *Lotus tetragonolobus* | Fucα1,3(Galβ4)GlcNAc |
| MAA I | *Maackia amurensis* agglutinin I | Gal (β-1,4) glcNAc |
| MAA II/MAH | *Maackia amurensis* agglutinin II | α2-3-linked sialic acids |
| MBL | Mannose binding lectin | fucose, mannose/mannan |
| MGL | Macrophage galactose-type lectin | Terminal α-or β-linked GalNAc |
| MMR | Macrophage mannose receptor | Terminal mannose, fucose or *N*‑acetylglucosamine |
| MOA | *Marasmium oreades* | Galα1 and 3Ga1 |
| MPA | *Macluria pomifera* | α-GalNAc (Tn-antigen), Galβ1-3GalNAc (T-antigen) |
| MNA-G | *Morus nigra* | Tn and Tα antigens |
| NPA | *Narcissus pseudonarcissus* | Terminal and internal Man |
| OAA | *Oscillatoria agardhii* | High-mannose-type glycan |
| PHA-E | *Phaseolus vulgaris-*Erythroagglutinin | Bisecting GlcNAc, biantennary N-glycans |
| PHA-L | *Phaseolus vulgaris*- Leucoagglutinin | Complex triantennary N-linked glycans |
| PNA | *Arachis hypogaea* | Galβ1-3 GalNAc (terminal) |
| PSA | *Pisum sativum* | α-Mannose |
| PTL-I | *Psophocarpus tetragonolobus-I* | α-GalNAc, Galα1-3 |
| PTL-II | *Psophocarpus tetragonolobus-II* | β-GalNAc |
| PWM | *Phytolacca americana* | Poly-LacNAc, GlcNAc oligomers |
| RCA | *Ricinus communis* | Gal-β1-4GlcNAc |
| RCA-I/RCA120 | *Ricinus communis* agglutinin | Galβ1-4GlcNAc, Galβ1-3Gal |
| RCA B | *Ricinus communis* agglutinin B | Terminal β-Gal, terminal LacNAc |
| RPL α-Gal | Recombinant prokaryotic lectin- α-Gal | α-Gal, GalNAc |
| RPL Gal-1 | Recombinant prokaryotic lectin- Gal-1 | β1,4-Gal LacNAc |
| RPL Gal-2 | Recombinant prokaryotic lectin- Gal-2 | α-Gal, GalNAc |
| RPL Gal-3 | Recombinant prokaryotic lectin- Gal-3 | α-Gal |
| RPL Gal-4 | Recombinant prokaryotic lectin- Gal-4 | β1,4-Gal LacNAc, Lewis^x^ |
| RPL Sia-1 | Recombinant prokaryotic lectin-Sia-1 | sialic acid |
| RPL Sia-2 | Recombinant prokaryotic lectin-Sia-2 | sialic acid |
| RPL Sia-3 | Recombinant prokaryotic lectin-Sia-3 | sialic acid |
| RPL Fuc-1 | Recombinant prokaryotic lectin-Fuc-1 | fucose |
| RPL Fuc-2 | Recombinant prokaryotic lectin-Fuc-2 | fucose |
| RPL Man-1 | Recombinant prokaryotic lectin-Man-1 | Mannose |
| RPL Man-2 | Recombinant prokaryotic lectin-Man-2 | Terminal Mannose |
| SBA | *Dolichos biflorus* | Terminal GalNAc |
| Siglec-2 | Sia-recognizing Ig-superfamily lectin 2 | Sia-> α6Gal-> β4GlcNac-> βR |
| Siglec-3 | Sia-recognizing Ig-superfamily lectin 3 | Sia-> α6Gal-> β4GlcNac-> βR |
| Siglec-5 | Sia-recognizing Ig-superfamily lectin 5 | Sia-> α3Gal-> β4GlcNac-> βR |
| Siglec-9 | Sia-recognizing Ig-superfamily lectin 9 | Sia-> α3Gal-> β4GlcNac-> βR, 6-sulfated sLe^x^ |
| Siglec-10 | Sia-recognizing Ig-superfamily lectin 10 | Sia-> α3Gal-> β4GlcNac-> βR |
| Siglec-11 | Sia-recognizing Ig-superfamily lectin 11 | Sia-> α8Sia-> α3Gal-> β4GlcNac  -> βR |
| SJA | *Sophora japonica* | GalNAc |
| SNA | *Sambucus nigra* | Sialic acid α (2-6) Gal |
| SSA | *Sambucus sieboldiana* | NeuAcα26Gal/GaINAc |
| STL | *Solanum tuberosum* | Poly-LacNAc, GlcNAc oligomers |
| SVN | Scytovirin | High Man |
| TJA-I | *Trichosanthes japonica* agglutinin-I | 6-Sulfo LacNAc or NeuAca2-6LacNAc |
| TJA-II | *Trichosanthes japonica* agglutinin-II | α 1-2Gal and β-GalNAc |
| TKA | *Trichosanthes kirilowii* | β-Gal, Neu5Acα2-3/6Galβ1 |
| TxLCI | *Tulipa gesneriana* | Manα1-3(Manα1-6)Man, bi/tri-antennary complex-type N-glycan, GalNAc |
| UDA | *Urtica dioica* | Poly-LacNAc, GlcNAc oligomers |
| UEA | *Ulex europaeus* | Fucα1-2Gal |
| VFA | *Vicia fava* | Man, Glc, GlcNAc |
| VGA | *Vicia graminea* | *O*-linked Galβ1-3GalNAc |
| VRA | *Vigna radiata* | α- and β -Gal |
| VVA | *Vicia villosa* | GalNAcβ1-4Gal, GalNAcβ1-3Gal, α-GalNAc |
| WFA | *Wisteria floribunda* | GalNAcα or β- 3 or 6 position of galactose |
| WGA | Wheat germ agglutinin (*Triticum aestivum*) | Terminal *N*-acetylglucosamine or chitobiose |

Man=mannose, GlcNAc = *N*-acetylglucosamine, GalNAc = *N*-acetylgalactosamine, LacNAc= *N*-acetyllactosamine, Sia = sialic acid, Fuc= fucose, Gal= galactose, Tn = T antigen, and novel sTn=sialyl lewis antigen
